# Microfluidics, 3D bioprinted scaffold and organ-on-chip with biosensing activated therapeutic strategies for diabetic wound healing

**DOI:** 10.3389/fbioe.2026.1865992

**Published:** 2026-08-07

**Authors:** Akanksha Jha, Abinaya R., Rajdeep Ojha, Manash K. Paul, Madhu Balaji Sivakumar, Suresh Ranga Rao, Tuhin Subhra Santra

**Affiliations:** 1 Department of Engineering Design, Indian Institute of Technology Madras, Chennai, India; 2 Department of Physical Medicine and Rehabilitation, Christian Medical College Vellore, Vellore, India; 3 Department of Radiation Biology and Toxicology, Manipal School of Life Sciences, Manipal Academy of Higher Education, Manipal, India; 4 Centre for Precision Medicine, Trichy SRM Medical College Hospital and Research Centre, Tiruchirappalli, Tamil Nadu, India; 5 School of Interdisciplinary Studies, Indian Institute of Technology Madras, Chennai, India

**Keywords:** biosensors, diabetic foot ulcer, microfluidics, organ-on-chip, point-of-care diagnostics, precision wound care, wound microenvironment

## Abstract

Diabetic foot ulcers (DFUs) represent one of the most severe complications of diabetes, arising from a complex systemic combination of neuropathy, vascular impairment, and impaired wound healing. These chronic wounds carry a high risk of infection, amputation, and mortality, which often leads to severe ulceration and amputation. Conventional diagnostic and monitoring strategies frequently fail to capture the dynamic microenvironment of DFUs, limiting timely therapeutic intervention. Recent advances in microfluidics and organ-on-a-chip technologies provide transformative opportunities for modelling DFUs, enabling precise recreation of the diabetic wound environment, including hypoxia, hyperglycaemia, inflammation, and microbial infection. Microfluidic wound models allow controlled study of cellular interactions, real-time monitoring of biochemical markers, and integration with biosensors for continuous assessment of glucose, lactate, pH, cytokines, and exosomal biomarkers. Furthermore, wearable and implantable microfluidic devices are emerging as platforms for non-invasive monitoring and personalized wound management. This review emphasizes the current state of the art in DFU pathophysiology, the clinical burden, and the limitations of existing management approaches, while highlighting the role of microfluidic and organ-on-chip technologies in advancing preclinical modelling, biosensing, therapeutic, and diagnostic development. By bridging engineering innovations with clinical needs, these technologies have the potential to revolutionize DFU research and pave the way for precision wound care strategies.

## 1 Introduction

**FIGURE 1:**
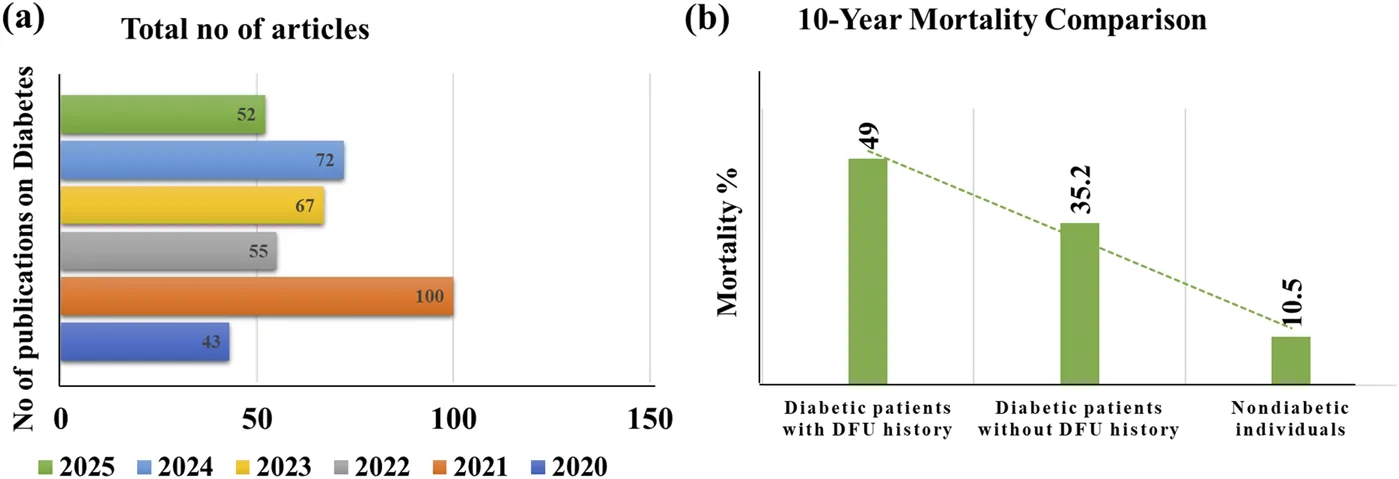
Analysis of publications on diabetes **(a)** Total number of research article published from year 2020–2025. **(b)** Comparison of mortality rates in diabetic patients suffering from diabetic foot ulcer (DFU) and patients without DFU. Data acquired as per Scopus in December 2025.

Diabetes is recognized globally as a significant public health concern. The highest global burden of diabetes is borne by India, with an estimated 100 million adults currently being affected, a number projected to substantially increase in the coming decades (Anjana et al., 2023). It is predicted that by 2025 the number of cases of diabetes in India would be around 69.9 million, with major cases being undiagnosed (Mathur et al., 2022). Globally, approximately 382 million individuals are affected by diabetes, and the continued rise in prevalence indicates that diabetes is likely to pose a major global pandemic challenge in the coming decades (Reed et al., 2021). The two broad classifications of diabetes mellitus are, Type 1 diabetes mellitus (T1DM), which is characterized by absolute insulin deficiency resulting from autoimmune-mediated destruction of pancreatic β-cells; and Type 2 diabetes mellitus (T2DM), which is a heterogeneous disorder defined by varying degrees of insulin resistance, impaired insulin secretion, and increased hepatic glucose production (Zhao et al., 2023). Among them T2DM accounts for the vast majority of diabetes cases in India (Pradeepa and Mohan, 2021). One of the most frequent and serious complications of this disease is the development of diabetic ulcers (Guo et al., 2024). DFU commonly occur as a consequence of neurological damage, vascular insufficiency, and biomechanical abnormalities. Studies indicate that 50%–60% of these ulcers progress to infection, and around 20% of moderate to severe cases eventually require lower limb amputation (Edmonds et al., 2021). The DFU is usually considered as deep tissue damage to the lower limb, and it occurs in 15% of people with diabetes, and the majority of them require amputation due to the risk of spread of infection in the bone and other related complications (Ojo et al., 2023). The long-term outlook is particularly concerning, as DFU’s are inherently chronic and progressive in nature; delayed healing, frequent recurrence, and stepwise clinical deterioration are commonly implicated, particularly in the midst of ongoing neuropathy, ischemia, and infection. Even after apparent wound closure, recurrence rates remain high, highlighting the relapsing course of diabetic foot disease and its substantial contribution to diabetes-related morbidity and mortality (Ingelfinger et al., 2017). Individuals with DFU face a 5-year mortality rate of about 30%, which escalates to nearly 70% following major amputations (Anbarasi et al., 2024). Mortality in this group remains alarmingly high, with approximately 231 deaths out of 1000 people per year recorded among those with ulcers, compared to 182 deaths out of 1000 persons per year in diabetic patients without ulcers (Chammas et al., 2016). Figure 1a shows the number of scientific articles related to diabetes published in the last 5 years and the comparison of the mortality rate of diabetic patients. Figure 1b shows mortality rate per year within two groups of diabetic patients one having foot ulcer and the other group without ulcers.

Uncontrolled and prolonged diabetes can cause ulceration, usually on the plantar surface of the foot, and can lead to chronic wound (CW) impairment. Three major pathologic mechanisms underlying are: neuropathy, impaired angiogenesis, and secondary infection due to trauma of the foot. The DFU results in debilitating osteomyelitis, deep soft tissue infection, gangrene, and amputation, hence it has a high level of morbidity. This makes it very important to treat the underlying cause (Raja et al., 2023). DFU harbour complex polymicrobial biofilms which contribute to adverse clinical outcomes with *Staphylococcus aureus* (*S. aureus*) is one of the most frequently identified pathogens (Heravi et al., 2019; Uberoi et al., 2024; Chen et al., 2026). Chronic infections may be caused by a mixed infection by several microorganisms, which also include gram-negative bacteria, anaerobes. The presence of this microbial network not only enhances antibiotic resistance but also interferes with host metabolic processes and further suppresses immune defence mechanisms, thereby compounding the severity of DFUs and impeding recovery (Bjarnsholt et al., 2018).

The management and healing of chronic DFU in patients have become a major therapeutic challenge (Naves, 2016). The healing process in diabetic wounds is severely hindered by multiple pathological factors, including persistent hyperglycaemia, tissue hypoxia, neuropathic complications, impaired angiogenesis, production of reactive oxygen species (ROS), and an increased vulnerability to microbial infections (Liang et al., 2017). Because of these challenges, timely identification and appropriate therapeutic intervention are vital to promote effective wound repair (Perez-Favila et al., 2019). At present, the principal treatment strategies for DFUs include surgical debridement to remove necrotic tissue, regular application of medicated dressings, stringent infection control measures, and negative pressure wound therapy (NPWT) as a supportive approach to accelerate healing (Balakrishnan et al., 2025).

Current therapeutic modalities support wound healing through various methods, but DFU treatment will be effective only if the disease is detected early and continuously monitored. In current practice, diagnosis of DFU is based on a multimodal assessment which includes clinical examination; testing for neuropathy using monofilament and bio-thesiometer-based vibration perception threshold measurements; vascular evaluation using ankle-brachial index and Doppler studies; radiographic imaging for suspected osteomyelitis; microbiological culture and sensitivity testing as well as assessment of plantar pressure, callus formation, foot deformities, and footwear (Donovan and Schweitzer, 2010). Despite their widespread availability, these testing modalities are episodic, fragmented, and poorly suited for dynamic wound monitoring (Kabir et al., 2023). Conventional treatment approaches for diabetic wounds often necessitate frequent hospital visits, which negatively impact patient quality of life, particularly in cases involving amputation, and impose substantial economic and financial strain (Serpico et al., 2023).

There has been rapid development in nano and microtechnology in the last decade integrated with biology, chemistry, biomedical, and mechanical engineering, a new era emerged: microfluidic and organ-on-a-chip devices capable of manipulating biological samples in a microenvironment with minimal sample consumption and contamination (Santra, 2020). Traditionally, *in vitro* disease studies intended to mimic human tissue in a controlled environment have failed to reflect the complexity of the *in vivo* system in several ways, including the absence of a physiological barrier, lack of cell−cell communication, and fluid-to-tissue ratios that differ from those in the human system (Li Lee, 2020). Compared to *in vitro*, microfluidics reduces excessive dilution of cell secreted factors hence leads to more cell-cell and cell media interactions. However, *in vivo* approaches are more tedious, laborious, and expensive, and does not generalize the effects on humans due to translational capacity of animal studies is impeded due to genetic differences (Marx et al., 2012). Microfluidic systems have significant advantages of wound healing and drug screening, this is achieved by precise drug control, minimally invasive monitoring, thus improving efficiency of *in-vitro* wound studies (Zhang and Staples, 2024). Microfluidic systems have been deployed in diagnosing and real-time monitoring of diabetes in the last 2 decades, which majorly focuses on glucose sensing, insulin in blood, and examination of the mechanical deformability of red blood cells (RBCs) (Liu et al., 2025a).

Microfluidic platforms present valuable opportunities for real-time monitoring and analysis of wound exudates by detecting critical biomarkers, including glucose concentrations, pH variations, and inflammatory mediators, such as interleukin-6 (IL-6) and tumour necrosis factor-alpha (TNF-α), which serve as indicators of infection and healing progression (Noushin et al., 2022). Beyond diagnostics, microfluidic and organ-on-a-chip technologies utilize integrated microfluidic channels to enable precise spatial and temporal delivery of antibiotics or growth factors, thereby promoting tissue repair and regeneration while minimizing systemic adverse effects. Consequently, the integration of microfluidics-based systems represents an innovative and promising strategy for the effective management of complex diabetic wounds (Zhao et al., 2022).

Deng et al. highlight chronic hyperglycaemia drives key pathological features such as peripheral neuropathy, vascular insufficiency, oxidative stress, and persistent inflammation, ultimately disrupting the normal wound healing cascade and leading to non-healing ulcers (Deng et al., 2023). Building on this foundation, Feng et al. encompasses also about conventional and advanced wound care strategies, including debridement, infection control, and biomaterial-based therapeutic interventions, while critically addressing their limitations in the diabetic microenvironment (Feng et al., 2025). Importantly, it integrates emerging biosensing platforms particularly flexible and microfluidics-based systems that enable real-time monitoring of wound biomarkers such as pH, glucose, oxygen, and inflammatory mediators, thereby facilitating precision-guided treatment. Furthermore, review by Phang et al. incorporates cutting-edge *in vitro* models, including 3D spheroid cultures and organ-on-chip (OOC) systems, which more accurately recapitulate the diabetic wound microenvironment and provide robust platforms for mechanistic studies and drug screening (Phang et al., 2021). By bringing together insights from pathophysiology, clinical wound management, biosensing innovation, and advanced tissue modelling, this review serves as a unified framework that bridges basic science and translational applications, aiming to advance the understanding and treatment of diabetic wound healing.

**FIGURE 2:**
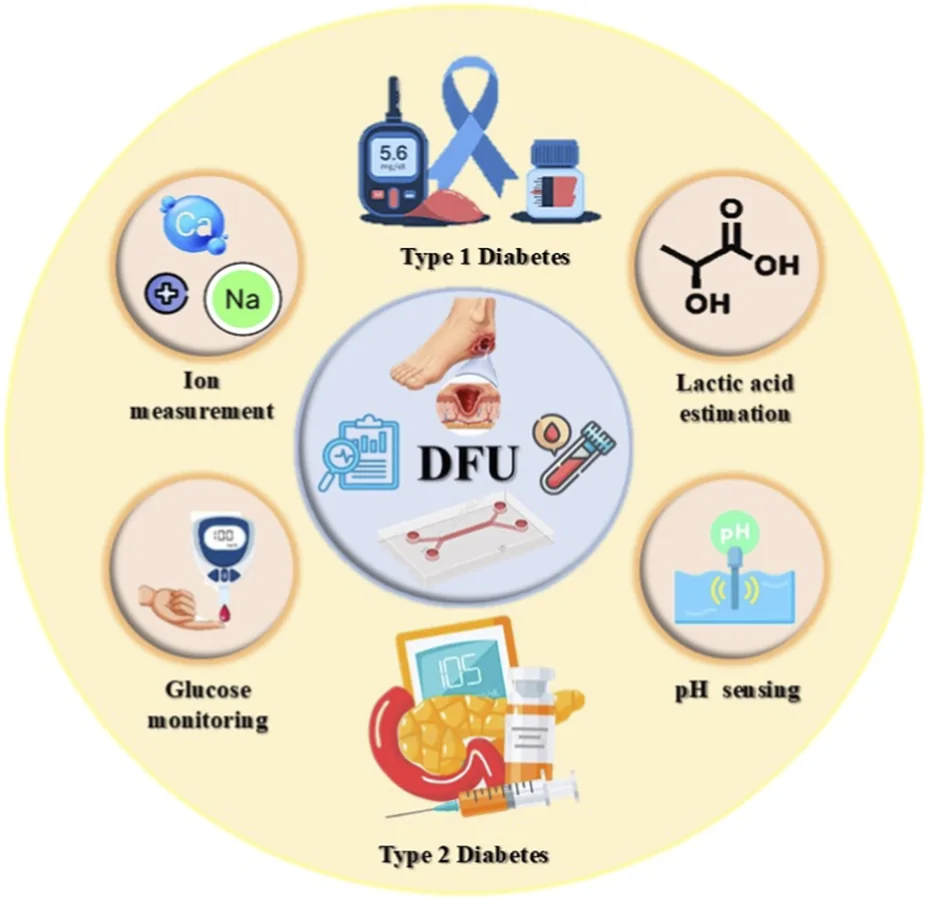
Schematic diagram representing microfluidics/organ-on-a-chip for diabetic wound healing.

Here, we review the current state-of-the-art microfluidic and organ-on-a-chip technologies for DFUs modelling, including hypoxia, hyperglycaemia, inflammation, and microbial infection. Microfluidic wound models help researchers study cellular interactions in a controlled environment. These systems also allow real-time monitoring of important biochemical markers. In addition, they can be integrated with biosensors for continuous measurement of glucose, lactate, pH, cytokines, and exosomal biomarkers. Furthermore, we addressed wearable and implantable microfluidic devices for non-invasive monitoring and personalized wound management. Figure 2 represents a schematic illustrating a microfluidic chip which can used for multimodal sensing to detect wound health for patients having DFU. This review presents a comprehensive and integrative perspective on DFUs by combining fundamental pathophysiological mechanisms with advances in wound management, biosensing technologies, and OOC approaches.

## 2 Pathophysiology

**FIGURE 3:**
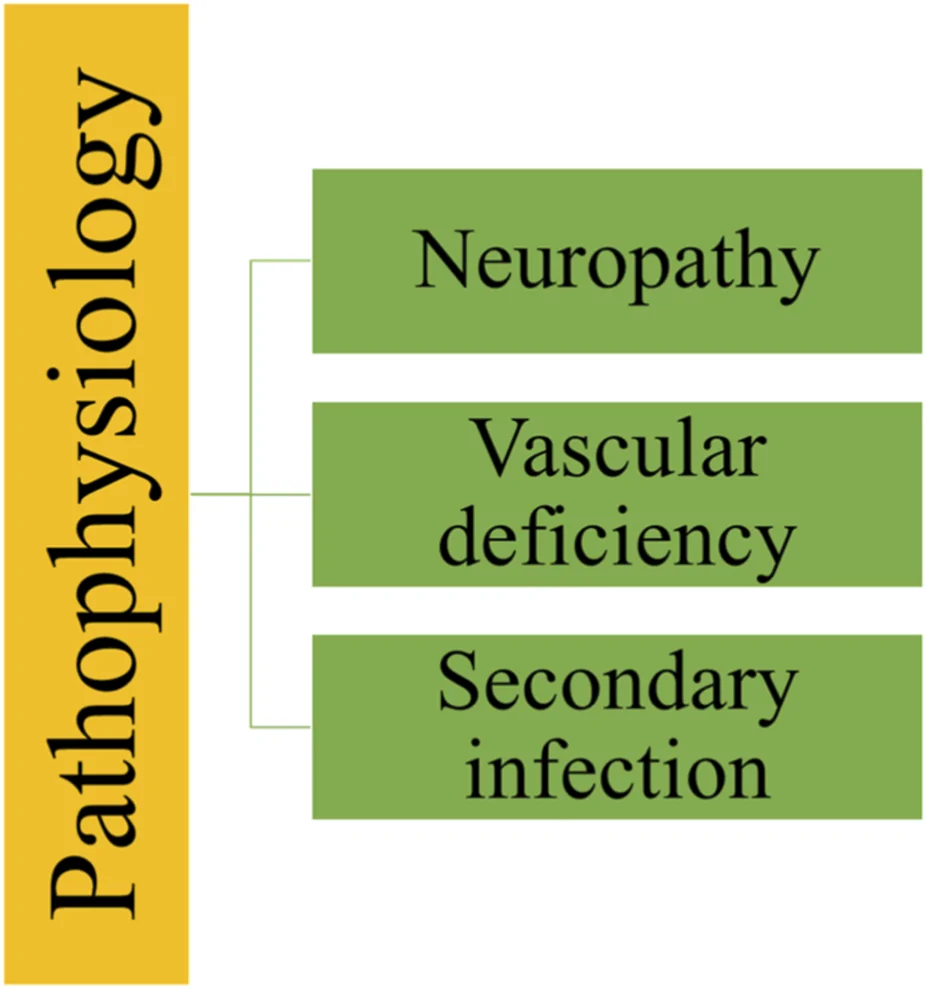
The triad of pathophysiological conditions in diabetes that leads to DFU.

The triad of pathophysiological conditions related to the DFU is neuropathy, secondary infection, and impaired angiogenesis (Syafril, 2018). Diabetes is usually not cause by only one pathophysiological condition, mostly the combined effect of these triad. Apart from the triad, several other factors also contribute to the condition. These include hyperglycaemia, hypoxia, prolonged inflammation, and reduced extracellular matrix solubility. In this section, we discuss how the three major conditions lead to disruption in wound healing and the imbalance of growth factors, cytokines, extracellular matrix (ECM) components, cell growth, and protease enzyme activity. Figure 3 illustrates the factors that cause diabetic foot ulceration.

### 2.1 Mechanism underlying impaired healing

#### 2.1.1 Neuropathy

Sensory impairment in individuals with diabetes is caused by hyperglycaemic circumstances, which result in upregulation of aldose reductase and sorbitol dehydrogenase which increases the sorbitol and fructose production (Alavi et al., 2014). The concentration of these glucose derivatives induces osmotic stress, reducing myoinositol synthesis in nerve cells and impairing neuronal transmission. In parallel, microvascular ischemia of the vasa nervorum, accumulation of advanced glycation end products, and oxidative stress–mediated mitochondrial dysfunction further accelerates axonal degeneration and nerve conduction failure (Tesfaye, 2010). In addition to sensory neuropathy, diabetes affects motor neurons, resulting in muscle atrophy and structural abnormalities in the foot (Raja et al., 2023). Motor neuropathy leads to muscle wasting and foot deformities, producing abnormal plantar pressure points. Collectively, these processes culminate in loss of protective sensation, which represents the key initiating factor for repetitive, unrecognized trauma and the subsequent development of DFU.

#### 2.1.2 Impaired angiogenesis

High blood sugar levels constrict blood capillaries, reducing the pressure gradient between the veins and the arteries. Endothelial dysfunction, oxidative stress, inflammation, and thickening of the capillary basement membrane collectively contribute to microvascular narrowing and impaired blood flow. This results in a reduced flow of blood through capillaries, reducing nutrition and oxygen supply (Yang et al., 2022). Effective wound healing requires an adequate blood supply to fulfil the heightened metabolic demands during the repair process. However, when this demand is not met, tissue regeneration is significantly impaired due to reduced nitric oxide (NO) bioavailability and impaired endothelial cell migration and proliferation (Tahergorabi and Khazaei, 2012). Insufficient vascular support also disrupts the timely transition of macrophages from the pro-inflammatory to the reparative phase, leading to excessive cytokine release, persistent neutrophilic activation, and increased fibroblast apoptosis (Wynn and Vannella, 2016). These events collectively extend the duration of the inflammatory stage, thereby further delaying the progression of wound healing (Feldman et al., 2019). Targeting the hyperglycaemic condition by administration of insulin and vascular endothelial growth factors (VEGF), which can restore vascularization. VEGF initiates angiogenesis and mediates endothelial growth and proliferation. The impaired blood flow, such as narrowed and blocked arteries, results in peripheral artery disease (PAD), which leads to low blood supply to the lower parts of the body, specifically the lower limbs (Soyoye et al., 2021). It can result in amputation due to accelerated atherosclerosis and impaired collateral blood vessel formation, which together exacerbate tissue ischemia, particularly in distal regions, making the condition more severe and difficult to reverse.

#### 2.1.3 Secondary infection and oxidative stress

CW has a slightly alkaline pH (pH = 8), which hinders the healing process (Wallace et al., 2019). Alkaline conditions elevate the activity of MMP (Matrix Metalloproteinase) protease, which causes the breakdown of structural proteins of the ECM (Tanase et al., 2025). As elevated MMP levels promote greater breakdown of ECM than ECM synthesis, this results in dysregulation of tissue repair. On the other hand, MMP activity is regulated by tissue inhibitors of metalloproteinases (TIMPs), which suppress MMP activity, but this equilibrium is disrupted by alkaline pH (Jones et al., 2015). Due to this change in pH, the growth factors activity and receptor signalling are affected, which makes the wound more vulnerable to secondary infection. The bioavailability and activity of key growth factors such as PDGF, TGF-β, and EGF are stable in mild acidic conditions, whereas in alkaline conditions it gets reduced. Alkalinity favours biofilm formation by enhancing bacterial adhesion. Thus pH-dependent changes in antibiotic ionization and bacterial membrane properties can reduce antibiotic uptake and activity. The altered microenvironment within the biofilm also promotes metabolically dormant bacterial cells, which exhibit increased tolerance to antimicrobial agents and reduced antibiotic penetration. Thus, an alkaline pH directly leads to infection persistence and chronicity (Dissemond et al., 2003).

### 2.2 Impaired wound healing in diabetes

Traditional wound healing is recognized as a highly intricate and tightly regulated physiological process aimed at restoring both the structural framework and functional integrity of the skin after injury (Kirsner and Eaglestein, 1993). Four overlapping phases are haemostasis, inflammation, proliferation, and remodelling. The haemostasis phase begins immediately upon tissue injury to prevent excessive blood loss (Singh et al., 2017). This phase is characterized by vasoconstriction and the activation of platelets, which aggregate at the site of the wound and initiate the coagulation cascade, in which exposure of tissue factor activates the extrinsic pathway, resulting in thrombin generation and the conversion of fibrinogen to fibrin (Green, 2006). When the platelets are trapped within the clot, they secrete a broad range of growth factors and cytokines that actively promote tissue repair. These include platelet-derived growth factor (PDGF), transforming growth factor-β1 (TGF-β1), epidermal growth factor (EGF), and thrombospondins (Bi et al., 2026). The inflammatory phase begins within hours of injury and is essential for the removal of damaged tissue and protection against infection. Neutrophils are the first responders, recruited via chemotactic signals such as interleukin-1β (IL-1β) and TNF-α (Reza Amini et al., 2020). These immune cells perform antimicrobial activities by engulfing pathogens through phagocytosis, producing ROS, and releasing proteolytic enzymes during degranulation. (Balakrishnan et al., 2025). Neutrophils also create neutrophil extracellular traps (NETs), which are made up of granular proteins, and chromatin, and aid in capturing and neutralizing pathogens. Fibroplasia, angiogenesis, and re-epithelialization are the processes that restore tissue structure during the proliferative phase (Landén et al., 2016). After being stimulated by PDGF and TGF-β1, fibroblasts migrate into the wound bed and release ECM components like fibronectin, and type III collagen. The last stage of wound healing is characterized by the maturation and structural reorganization of the newly generated tissue. In this phase, fibroblasts differentiate into myofibroblasts enabling wound contraction by drawing the ECM together, thereby decreasing the overall wound area (Cialdai et al., 2022). Wound healing Section S.1 is added in detail in Supplementary Material.

**FIGURE 4:**
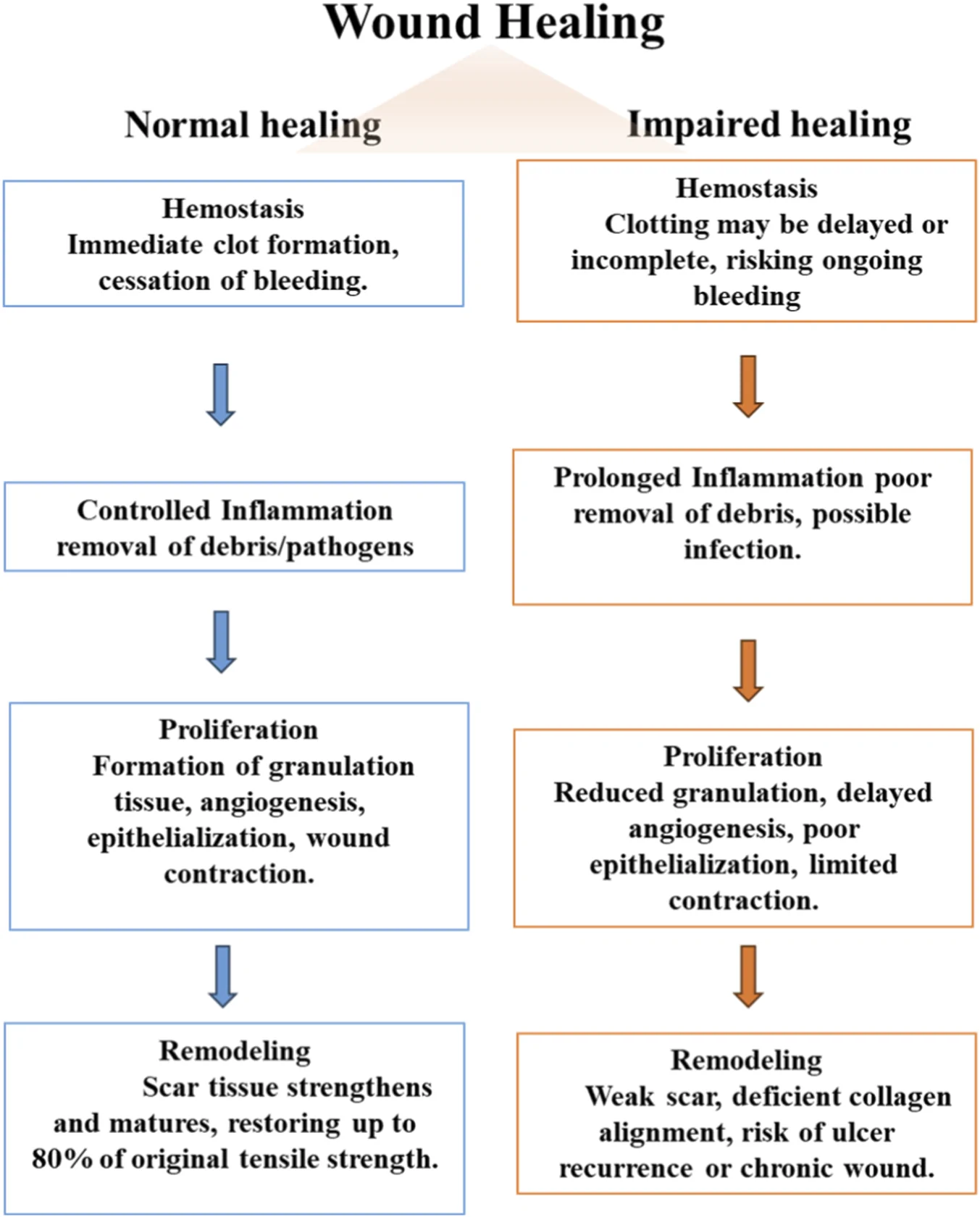
Illustration on the key differences between normal and impaired wound healing processes. The four stages of healing Haemostasis, inflammation, proliferation and remodelling are highlighted.

The wound healing process in individuals with diabetes is profoundly compromised due to an interplay of complex systemic metabolic dysregulation, disrupted angiogenic process, less endothelial progenitor cells, and an imbalance in ECM regulation (Spampinato et al., 2020). While traditional wound healing proceeds in an ordered sequence of haemostasis, inflammation, proliferation, and remodelling, diabetic wounds often fail to transition through these phases in a timely and coordinated manner. This results in the development of chronic, non-healing ulcers that are susceptible to infection, tissue necrosis, and gangrene, which leads to limb amputation if not managed effectively. Figure 4 shows how wound healing differs at different stages in diabetic patients in comparison to normal wound healing.

#### 2.2.1 Prolonged inflammation and impaired immune response

In diabetic patients, an indication of impaired wound healing is prolonged inflammation. Hyperglycaemia-induced oxidative stress and advanced glycation end products (AGEs) lead to chronic activation of immune pathways, resulting in sustained infiltration of neutrophils and macrophages at the wound site (Xiong et al., 2025). Immune cells persistently secrete pro-inflammatory cytokines such as IL-1β and TNF-α, along ROS, which collectively contribute to establishing a hostile, inflammatory wound microenvironment (Nirenjen et al., 2023). In diabetic wounds, the usual shift of macrophages from the pro-inflammatory M1 state to the reparative M2 state is disrupted, thereby hindering healing progression and delaying the transition into the proliferative phase (Balakrishnan et al., 2025).

Additionally, diabetic patients exhibit compromised neutrophil and macrophage function, including reduced phagocytosis and chemotaxis, which diminishes the clearance of pathogens and cellular debris. This increases susceptibility to infection and contributes to chronic inflammation (Nigi et al., 2025).

#### 2.2.2 Impaired angiogenesis and hypoxia

Effective wound healing requires robust neovascularization to supply nutrients and oxygen to regenerating tissue. In diabetic wounds, angiogenesis is significantly impaired due to the downregulation of key pro-angiogenic factors such as VEGF and angiopoietin-1, coupled with endothelial cell dysfunction characterized by downregulation and impaired signalling of VEGF receptors. Resulting in attenuated angiogenic responses even when VEGF is present (Kolluru et al., 2012). Chronic hyperglycaemia suppresses eNOS (endothelial NO synthase) activity indirectly leading to a decrease in NO production, which is critical for the migration of endothelial cells and the formation of blood vessels (Okonkwo et al., 2020). Furthermore, decreased mobilization and poorer anchoring of endothelial progenitor cells (EPCs) at the site of the wound contribute to poor vascularization and persistent tissue hypoxia (Zubair and Ahmad, 2019).

#### 2.2.3 Defects in fibroblast activity and ECM remodelling

Fibroblast dysfunction is another key feature of impaired diabetic wound healing. Fibroblasts differentiate into myofibroblasts, and these play an essential role in the proliferation and remodelling phase of the wound healing process (Wan et al., 2021). High glucose levels inhibit fibroblast proliferation and migration, leading to insufficient ECM production and delayed granulation tissue formation (Schultz and Wysocki, 2009). Additionally, chronic wounds in diabetes show altered function and expression of MMPs and their inhibitors. Excessive MMP activity leads to the degradation of essential ECM components, such as collagen and fibronectin, while reduced TIMPs activity fails to restore matrix balance (Ayuk et al., 2016). This unregulated proteolytic environment impedes tissue regeneration and epithelial migration.

#### 2.2.4 Keratinocyte dysfunction and delayed re-epithelialization

Re-epithelialization, which is driven by keratinocyte proliferation and migration from wound edges, is significantly delayed in diabetic wounds (Mansoub, 2021). This is partly due to reduced keratinocyte responses to EGF and insulin-like growth factor (IGF), both of which are downregulated in diabetics. Glycation of growth factors, such as fibroblast growth factor-2 (FGF-2), further reduces their capacity to bind receptors and activate downstream signalling pathways required for wound closure (Zheng et al., 2023). Moreover, the diabetic wound environment often lacks the appropriate integrin signalling and cytoskeletal reorganization required for efficient keratinocyte migration.

#### 2.2.5 Microvascular and neuropathic complications

Peripheral arterial disease and diabetic neuropathy play critical roles in delaying wound healing (Tesfaye, 2010). Microvascular dysfunction results in reduced capillary perfusion, oxygen delivery, and nutrient transport. Basement membrane thickening and endothelial cell abnormalities impair oxygen diffusion, contributing to sustained hypoxia at the wound site (Vithian and Hurel, 2010). Neuropathy exacerbates these effects by impairing neurogenic control of blood flow and reducing the pain response, which increases the likelihood of unnoticed trauma and repetitive injury.

#### 2.2.6 Alkaline pH and oxidative stress

Diabetic wounds often exhibit an alkaline pH, which enhances protease activity including MMPs, and further disrupts ECM integrity (Jones et al., 2015). Excessive production of ROS and the formation of an alkaline wound microenvironment contribute significantly to persistent bacterial colonization in chronic wounds such as diabetic foot ulcers. While physiological ROS levels support antimicrobial defence, cell signalling, and tissue repair, chronic hyperglycaemia and prolonged inflammation lead to excessive ROS generation through mitochondrial dysfunction and AGE-mediated pathways. Elevated ROS levels cause oxidative damage to cellular components, impair fibroblast and keratinocyte function, reduce angiogenesis, and disrupt ECM remodelling. These changes weaken local immune defence and create a favourable environment for bacterial persistence and delayed wound healing. These conditions favour bacterial colonization, leading to biofilm formation and thereby increasing the risk of persistent infections.

## 3 Types of wound healing models

### 3.1 Wound models

A wound-healing study employs a range of experimental models to simulate the physiological processes involved in tissue repair (Low et al., 2021). Chronic wound healing remains a critical clinical challenge, prompting the development of diverse experimental models to understand delayed-healing mechanisms and evaluate therapeutic strategies (Tan et al., 2023). Unlike acute wounds, which progress through coordinated phases of haemostasis, inflammation, proliferation, and remodelling, chronic wounds exhibit impaired or delayed healing due to conditions such as diabetes mellitus, vascular insufficiency, prolonged inflammation, and excessive ROS production, which collectively disrupt cellular and tissue integrity (Flynn et al., 2023). These models are typically categorized into *ex vivo, in vivo, in vitro,* and increasingly, human clinical models. Each model type plays a critical role in understanding the cellular mechanisms, testing therapeutics, and translating basic research findings into clinical applications.

#### 3.1.1 *In Vitro* models

*In vitro* wound healing models provide simplified, controlled environments for studying cellular behaviours such as migration, proliferation, and cytokine expression (Dewangan et al., 2017). The most common method, the scratch assay, involves mechanically creating a wound in a cell monolayer typically fibroblasts, keratinocytes, or endothelial cells and observing cellular migration into the void (Cappiello et al., 2018). Co-culture systems and 3D skin equivalents (constructed from collagen matrices seeded with multiple skin cell types) have further improved the physiological relevance of *in vitro* systems. These models are ideal for high-throughput drug screening, toxicological assessments, and mechanistic studies. However, their lack of vasculature, immune cell infiltration, and systemic regulatory mechanisms limits their utility in mimicking the complexity of real wound healing responses (Low et al., 2021). *In vitro* wound models fail to replicate key biophysical and biochemical features of the wound microenvironment, including mechanical forces, ECM stiffness, and dynamic gradients of oxygen, glucose, and cytokines (Xiao et al., 2017). Similarly, 3D bioprinted chronic wound constructs, although promising, remain technically complex and lack fully functional vasculature and innervation. 3D bioprinted chronic wound constructs embedded with fibroblasts and immune cells from chronic wound biopsies (Greenhalgh, 2005) are usually static and lack temporal regulation, limiting their ability to model phase-specific healing responses or chronic wound states such as those observed in diabetes. Reproducibility is also a concern, particularly in scratch assays, where variability in wound width, edge damage, and cell injury can influence experimental outcomes.

#### 3.1.2 *Ex vivo* wound models

*Ex vivo* offers a more physiologically accurate alternative to *in vitro* systems by using full-thickness human or animal skin excised and maintained under perfused or static conditions (Ueck et al., 2017). These models preserve the multicellular architecture of native skin, including epidermal, dermal, and ECM components. *Ex vivo* systems are particularly useful for testing wound dressings, antimicrobial agents, as well as studying scar formation, mechanistic studies, controlled experiment conditions and drug toxicity and penetration. Their use has expanded into specialized settings, such as keloid modelling, where human scar tissue is maintained *ex vivo* to study photodynamic therapies or anti-fibrotic drugs (Wilhelm et al., 2017). Furthermore, donor-to-donor variability, limited tissue availability, and ethical issues limit scalability and reproducibility.

#### 3.1.3 *In vivo* wound models

*In vivo* wound healing models are indispensable in preclinical research because they allow a comprehensive assessment of wound repair, integrating immune responses, angiogenesis, systemic metabolism, and tissue remodelling (Ahmad, 2023). Rodent models, particularly mice and rats, are widely employed to study wound healing, including full-thickness excisional wounds, incision models, burn injuries, and diabetic wound conditions (Sanapalli et al., 2021). These systems are valuable for evaluating anti-inflammatory, angiogenic, and re-epithelialization therapies in a time-dependent manner (Ahmad, 2023). Pigs are frequently used for translational studies because their skin is anatomically and physiologically similar to humans, including dermal thickness, collagen distribution, and healing patterns (Flynn et al., 2023). However, ethical concerns, high costs, and interspecies variability remain important limitations. Therefore, findings from these models should be interpreted cautiously before clinical application. Rodents predominantly heal wounds through contraction mediated by the panniculus carnosus, a mechanism that differs substantially from that in humans, which relies primarily on re-epithelialization (Naldaiz-Gastesi et al., 2018). Moreover, commonly used diabetic animal models fail to fully recapitulate the chronic metabolic, vascular, and neuropathic complications characteristic of human diabetic foot ulcers. Species-specific immune responses and limited control over local wound microenvironmental parameters further constrain translational predictability. In addition to animal-based models, humanized models are gaining popularity due to their translational value. Porcine chronic wound models are considered highly relevant due to their close resemblance to human skin in dermal–epidermal thickness, immune response, and healing mechanisms. Unlike rodents, pigs exhibit minimal wound contraction and predominantly heal through re-epithelialization, making them valuable for preclinical studies of chronic wounds such as diabetic foot ulcers and pressure ulcers (Hadad et al., 2010). Chronic wound conditions in porcine models can be induced through ischemia, biofilm formation, repeated trauma, irradiation, or impaired perfusion. In addition, the absence of panniculus carnosus and minimal wound contraction in pigs make these models suitable for studying re-epithelialization and neovascularization. However, their widespread application is limited by high maintenance costs, specialized housing requirements, and lower genetic tractability. The Lanyu pig model is particularly useful for distinguishing full-thickness and partial-thickness healing dynamics, where impaired rete ridge regeneration in full-thickness wounds resembles scar-prone chronic human wounds (Lin et al., 2019a).

**FIGURE 5:**
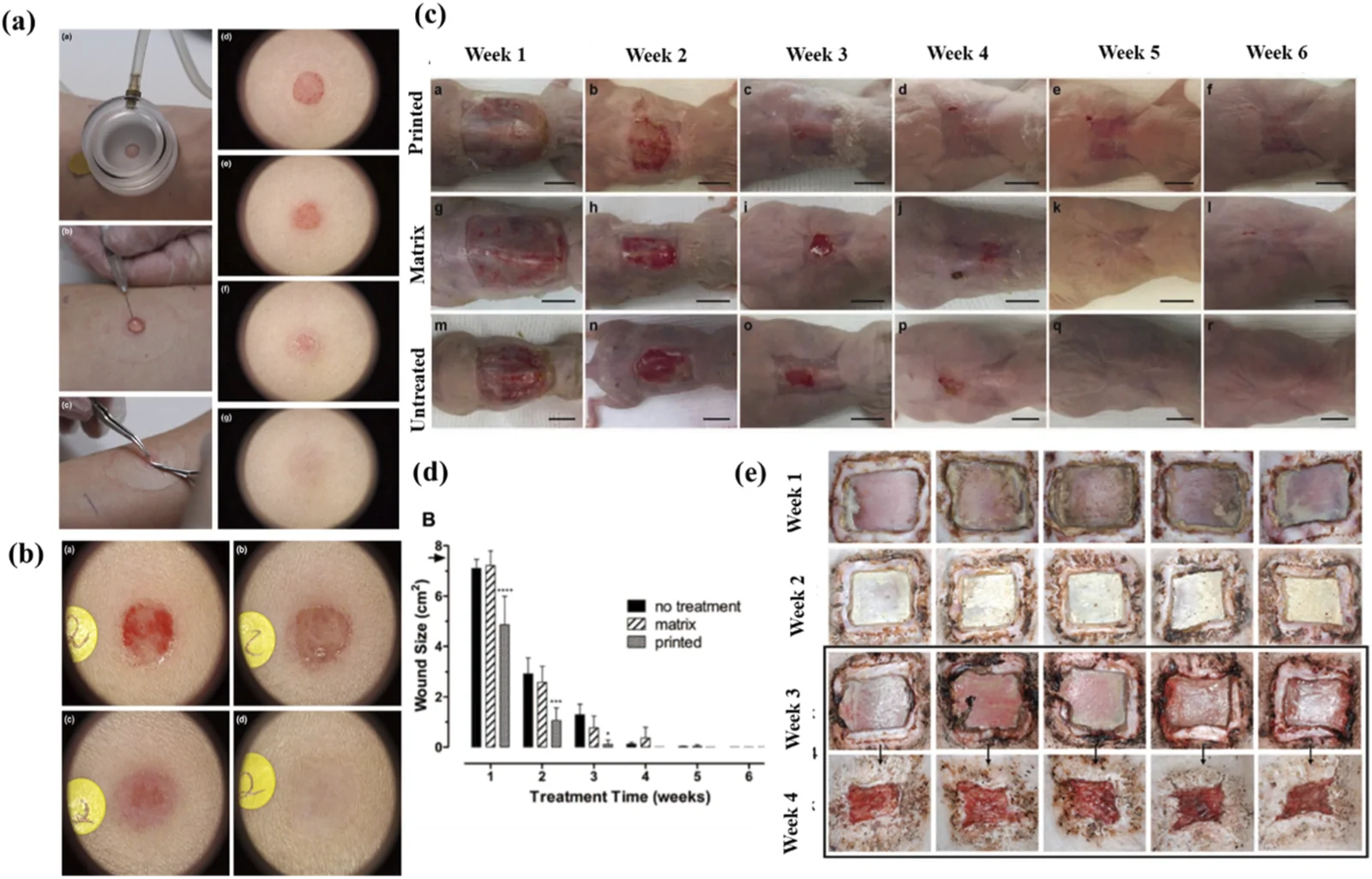
**(a)** Suction blister model by applying a vacuum of 300 mmHg for 3–4 h by suction cups with an opening diameter of 4–8 mm. Reproduced with permission, copyright 2016, Wiley (Wilhelm et al., 2017). **(b)** Abrasive wound model study for day 0, day 3, day 5 and day 15. Reproduced with permission, copyright 2016, Wiley (Wilhelm et al., 2017). **(c)** Cell printed mice show epithelium forming over the wound as early as week 1, with developing skin observed at week two that covers the entire wound but has not fully formed. By week 3, cell printed mice show complete coverage of the wound. Between week 4 and week 6, minimal contraction is observed and we observe a maturing epithelium. In contrast, matrix-treated and untreated wounds show minimal epithelialization until week 4, resulting in a significant proportion of open wound area. Contraction is also significant between weeks 4 and 6 following closure of the wounds. Scale bar: 1 cm. Reproduced with permission, copyright 2019, nature (Albanna et al., 2019). **(d)** Analysis of murine wound sizes over 6 weeks. Printed skin constructs show a significantly reduced time to wound closure when compared to untreated and matrix-treated controls. Printed skin closed the wound in 3 weeks compared to 5 weeks for controls. Wound sizes were analyzed with one-way ANOVA. ****p < 0.0001, n = 12; ***p < 0.01, n = 8; *p < 0.05, n = 8. Data presented as mean ± standard deviation (SD). Reproduced with permission, copyright 2019, nature (Albanna et al., 2019). **(e)** porcine experimental wound model, which had a silicone block till 3 weeks of surgery. Reproduced with permission, copyright 2012, Wiley (Jung et al., 2013).

Figure 5c represents epithelium formation for week 1 to week 6 in cell printed mice. Figure 5d shows analysis of murine wound sizes over 6 weeks. Printed skin constructs show a significantly reduced time to wound closure when compared to untreated and matrix-treated controls (Albanna et al., 2019). Figure 5e shows images of experimental wounds. In this study epithelialisation and wound contraction were not detected in the porcine model while silicone blocks were inserted. Following 3 weeks of surgery, the silicone blocks were removed. Wounds were largest at 3 weeks. Silicone blocks were removed at 3 weeks after surgery, and rapid wound epithelialisation and contraction followed. Nonetheless, wound healing was far from complete at 4 weeks after surgery (Jung et al., 2013).

#### 3.1.4 Human wound healing models

It represents the gold standard for translational relevance. These include both clinical wound studies in patients and controlled human volunteer wound models. In such models, small incisions, excisions, or suction blisters are created under ethical approval to study acute healing responses, wound dressings, or novel therapeutics in a human biological context (Wilhelm et al., 2017). These models offer unparalleled relevance for understanding wound healing kinetics, scar formation, and pain perception in human skin. Additionally, wound biopsies obtained from patients with chronic wounds (such as venous ulcers or diabetic foot ulcers) are used to study delayed-healing processes and test regenerative therapies in real-world pathological contexts (Mojumdar et al., 2021). However, its application is limited by legislative, ethical, and logistical constraints, as well as by individual heterogeneity. These systems provide an opportunity to replicate the biochemical and biomechanical microenvironment of chronic wounds, including fibrosis, bacterial colonization, and senescent cell accumulation (Wang and Shi, 2020). Additionally, human volunteer models such as suction blister or tape-stripping injury allow controlled investigation of early wound healing in pathological settings (e.g., in diabetic patients), although ethical limitations restrict the depth and chronicity, that can be studied.

Human models are inherently low-throughput and unsuitable for studying long-term wound chronicity. Collectively, these issues highlight the need for complementary platforms that combine human relevance with experimental control and scalability. Figures 5a,b show the suction blister model and abrasive wound model, which were studied by Wilhelm et al. In this study, wounds of varying depths were created in human subjects, and their healing was studied. The results showed proper healing without scarring (Wilhelm et al., 2017).

#### 3.1.5 In silico modelling

Moreover, emerging technologies, such as *in silico* modelling, are increasingly used to simulate chronic wound progression and predict outcomes of therapeutic interventions (Ud-Din and Bayat, 2017). These computational approaches incorporate patient-specific parameters such as age, comorbidities, and inflammatory profiles to provide a virtual testing ground for wound therapies (Flynn et al., 2023). They serve as a bridge between reductionist models and complex human pathology, reducing experimental burden and enhancing predictive power. However, their utility depends heavily on the quality of the experimental and clinical data used for modelling, and thus, they are not standalone solutions. In addition, current computational algorithms often rely on simplified biological assumptions and lack the capacity to fully recapitulate dynamic cellular interactions, ECM remodelling, angiogenesis, and host–pathogen responses characteristic of chronic wounds (Crossley et al., 2024).

### 3.2 Wound management

**FIGURE 6:**
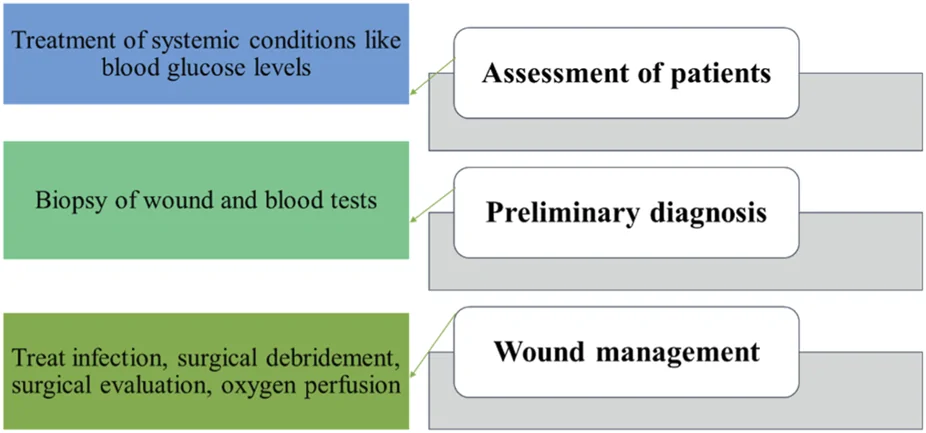
The process steps that are included in the management strategy for treating diabetic foot wounds.

Effective management of chronic wounds is a multifactorial challenge that requires an integrated approach combining accurate diagnosis, etiological treatment, and appropriate wound care. Figure 6 shows the process steps of advanced or adjunctive therapies of wound management (Powers et al., 2016). Chronic wounds, including diabetic foot ulcers, venous leg ulcers, and pressure injuries, exhibit impaired healing characterized by persistent inflammation, poor perfusion, infection, and complications associated with systemic disorders such as diabetes and vascular insufficiency (Xiang et al., 2019). The primary objective of chronic wound management is restoration of the wound’s physiological environment, thereby reinitiating the stalled healing process.

A foundational component of wound care is wound bed preparation, which includes the systematic removal of nonviable tissue (debridement), control of inflammation, infection, moisture balance, and the stimulation of the wound edge collectively referred to as the tissue management, inflammation/infection control, moisture balance, and edge of wound advancement which is the TIME principle (Schultz et al., 2004). Debridement whether surgical, enzymatic, autolytic, or mechanical such as wound washing and scrubbing of the wound plays a critical role in reducing bioburden and removing necrotic tissue that otherwise supports biofilm formation and chronic inflammation. Particularly in diabetic and venous ulcers, repeated debridement has been shown to stimulate granulation tissue formation and improve healing rates.

The benefits of this were explored in the literature, where moist wounds show significantly better quality of healing (Nuutila and Eriksson, 2021). The NPWT enhances wound contraction, eliminates exudate, and improves perfusion by applying sub-atmospheric pressure to the wound bed, thereby stimulating cell proliferation and angiogenesis (Tang et al., 2023). This approach has shown great potential in diabetic foot ulcers and significant pressure injuries, when traditional methods fail (Kavitha, 2014).

For non-healing or complex wounds, advanced therapies are introduced. These include growth factor therapies (e.g., PDGF, VEGF), bioengineered skin substitutes, stem cell therapy, and gene therapy (Sharma et al., 2024). Although these therapies are promising in experimental models their clinical efficacy is often hindered by the complex chronic wound environment, which degrades growth factors rapidly or impairs cell viability. Autologous platelet-rich plasma (PRP) and mesenchymal stem cells (MSCs) have sparked interest due to their immunomodulatory and angiogenic properties. However, such therapies are costly, necessitate expert handling, and have yet to demonstrate consistent effectiveness across patient populations in large-scale studies (Yazdanpanah, 2015).

In recent years, technologies such as wound imaging, telemedicine, and artificial intelligence-assisted wound assessment tools have been integrated into wound management practices (Yao et al., 2013). These innovations improve documentation, facilitate early intervention, and support personalized treatment decisions, particularly in rural or resource-limited settings. In addition to advanced wound-monitoring technologies, effective management of chronic wounds also requires coordinated clinical care teams involving dermatologists, podiatrists, specialists for infectious disease, vascular surgeons, and wound nurses have been shown to improve outcomes by ensuring comprehensive care and continuity. A microfluidic device, that can sense the wound conditions with different parameters such as pH and chemical complexity and transfer the data to the clinician in real time, will help improve the wound management (Wang et al., 2024a).

### 3.3 Wound classification

Wound classification plays a pivotal role in the management and prognosis of DFUs. Systematic and accurate classification is essential for guiding treatment, monitoring effectiveness, predicting outcomes, and enabling clear communication among healthcare professionals. Patel et al., explores multimodal basis of classification by using image processing and a deep neural network by concatenating the image-based and the classification-based datasets (Anisuzzaman et al., 2022). The studies provided underscore the clinical relevance of wound classification systems and describe in detail the most prominent systems used for diabetic foot ulcers: Wagner and the University of Texas (UT) systems (Santema et al., 2016).

The necessity for a standardized, reliable, and easy-to-use wound classification system is rooted in several critical needs, such as planning treatment, which aids in devising tailored strategies for an ulcer with infection and ischemia, that might require urgent intervention. Secondly, with the help of a wound classification system, monitoring and communication over time help in advanced patient care (Gallagher et al., 2024). The next study proposed shows that prediction based on the classification system helps us to predict the likelihood of the amputation, and the study provides a common ground for evaluation and treatment approaches (Liu et al., 2025b). The severity of the ulcer has been studied in patients and is clinically classified according to Wagner-Meggitt classification. The severity of the ulcer was graded from 0 to 1 (Table 1). However, to correlate severity with the presence of ischemia or infection, it can refer to the University of Texas classification, which grades severity from 0 to 3 (Table 2) (Niță et al., 2023).

**TABLE 1:** Wagner-Meggitt classification of diabetic foot ulcers.

Wagner’s grade	Characteristics
Grade 0	Intact skin
Grade 1	Superficial skin
Grade 3	Abscess or osteomyelitis
Grade 4	Forefoot gangrene
Grade 5	Whole foot gangrene

**TABLE 2:** University of Texas (UT) classification of diabetic foot ulcer.

Grade Stage	Grade A	Grade B	Grade C	Grade D
Stage 0	Epithelialized + No infection and No ischemia	Epithelialized + Infection	Epithelialized + Ischemia	Epithelialized + Infection and Ischemia
Stage I	Superficial wound + No infection and No ischemia	Superficial wound + Infection	Superficial wound + Ischemia	Superficial wound + Infection and Ischemia
Stage II	Wound penetration to tendon + No infection and No ischemia	Wound penetration to tendon + Infection	Wound penetration to tendon + Ischemia	Wound penetration to tendon + Infection and Ischemia
Stage III	Wound penetration to bone + No infection and No ischemia	Wound penetration to bone + Infection	Wound penetration to bone + Ischemia	Wound penetration to bone + Infection and Ischemia

#### 3.3.1 Wagner wound classification system

The Wagner system is one of the earliest and most widely adopted diabetic foot ulcer classification. It grades ulcers from 0 (pre- or post-ulcerative) to 5 (whole foot gangrene), primarily depends on anatomical depth and the absence or presence of osteomyelitis and gangrene (Mehraj, 2018). Table 1 shows Wagner-Meggitt classification of diabetic foot ulcers.

#### 3.3.2 University of Texas (UT) wound classification system

The UT system was developed to address the limitations of the Wagner system by offering a more detailed and practical approach. The UT system classifies wounds according to both grade (depth) and stage (presence of infection and/or ischemia) (Santema et al., 2016).

### 3.4 Clinical consequences in DFU progression

The progression of DFUs triggers a cascade of clinical consequences that affect limb preservation, quality of life, and survival rates. Below is a detailed description of these consequences, integrating foundational literature, cohort studies, meta-analyses, and recent research findings. Foot ulcers represent a significant health burden for diabetic patients, being a common precursor to amputation and often leading to increased morbidity and mortality. According to research by McDermott et al., ulceration affects 5%–10% of the diabetic population, with up to 3% requiring lower limb amputations (McDermott et al., 2022). Infection, peripheral vascular disease, and increased wound depth all raise the risk of poor clinical outcomes. The presence of neuropathy, ischemia, and infection compounds further leads to delayed healing (Lombardi et al., 2026).

#### 3.4.1 Escalating risk of infection and severe complications

The DFU progression creates a persistent breach in the skin barrier, which invites bacterial colonization. Initial infections are often caused by aerobic Gram-positive cocci, particularly *S. aureus*, but chronic or deep ulcers can rapidly become polymicrobial, incorporating Gram-negative and anaerobic pathogens (Kwon and Armstrong, 2018). Pathogens such as *Pseudomonas aeruginosa* (*P. aeruginosa*) and methicillin-resistant *Staphylococcus aureus* (MRSA) are particularly difficult to eradicate due to their propensity for biofilm formation and their intrinsic antimicrobial resistance, which contribute to persistent infections and delayed wound healing. Advanced infection can penetrate deeper layers, resulting in soft tissue abscess, necrotizing fasciitis, and frequently osteomyelitis (bone infection), further complicating healing and often requiring complex surgery or extended antibiotic therapy (Raja et al., 2023). If inadequately treated, diabetic foot ulcer (DFU) infections can progress to systemic infection and sepsis, significantly increasing the risk of morbidity and mortality (Fitrianingsih et al., 2025).

#### 3.4.2 Progressive ischemia, necrosis, and gangrene

Tissue ischemia and necrosis are commonly associated with peripheral arterial disease, involved in half of all cases. Disease progression hinders healing and encourages necrosis (Lin et al., 2022). When wound healing is further derailed by infection and vascular insufficiency, tissue death (gangrene) may occur, especially in severe or untreated cases. Gangrene and irreversible tissue loss are severe late-stage complications observed in Wagner grade 4 and 5 ulcers, often necessitating partial or complete foot amputation to control infection and prevent life-threatening complications (Elgzyri et al., 2021).

#### 3.4.3 Increased likelihood of amputation

DFU are a major cause of lower-limb amputations worldwide. Minor amputations, involving the removal of toes or parts of the foot, are commonly performed in severe cases. In dedicated multidisciplinary care settings, healing following below-ankle amputations, including auto-amputation or minor amputation, can be achieved; however, the healing process is often prolonged (Lin et al., 2020). Whereas in major amputation, the progression to extensive necrosis, severe unresponsive infection, or nonviable tissue may require amputation above the ankle. Major amputation is reported in 16%–22% of cases and carries very high morbidity, and post-amputation mortality is caused predominantly by cardiovascular events, infections, renal comorbidities, and progressive functional decline, with reported 5-year mortality rates exceeding about 50% (Elgzyri et al., 2021). Recent meta-analyses estimate global lower-limb amputation rates among DFU patients at 22%–31%, with higher rates in advanced ulcer grades or those with peripheral vascular disease present (Zhang et al., 2024).

## 4 Emerging advanced therapies for DFU

### 4.1 Bioengineered skin substitutes and tissue-engineered scaffolds

Bioengineered skin and tissue-engineered methods signify an advancement in wound care, offering innovative options for addressing both chronic and acute wounds that often pose difficulties for current treatments (Kondej et al., 2024). These advanced techniques focus on repairing or replacing injured skin by integrating biological, chemical, and physical sciences to create skin substitutes and scaffolds that replicate the architecture and functionality of natural tissue (Tavakoli and Klar, 2021). Chronic wounds, such as DFU, burns, and pressure sores, present significant clinical challenges due to impaired healing and a high risk of infection. Conventional dressings often fail to provide the necessary biological cues or support for complete tissue regeneration. As such, there is a growing emphasis on bioengineered constructs that not only act as physical barriers but also actively participate in the healing process, delivering cells, growth factors, and bioactive molecules to the wound environment (Kondej et al., 2024). Bioengineered skin substitutes are fabricated using a combination of synthetic and natural biomaterials, living cells such as endothelial cells, keratinocytes, fibroblasts, and tailored architectures that promote vascularization, cell migration, and tissue integration (Lu and Huang, 2012). Tissue-engineered scaffolds function as temporary extracellular matrices, guiding the repair and regeneration of native skin by providing structural support and modulating cellular behaviour. Advances in biomaterial science, 3D bioprinting, and stem cell technology have enabled the creation of highly functional wound dressings that closely resemble native skin in both appearance and performance (Kyriakidis et al., 2021).

Nanofibrous scaffolds, typically fabricated by electrospinning, provide high surface area and ECM-like architecture, supporting cell adhesion and controlled drug release. Their tunability makes them attractive for diabetic wound applications; however, large-scale reproducible fabrication and effective integration within dynamic wound environments remain significant challenges (Martins et al., 2007). In parallel, ECM-mimicking scaffolds aim to replicate the biochemical and biomechanical properties of human skin, while tissue-engineered substitutes and biomaterial dressings, including living skin equivalents such as Apligraf and DermaGraft, as well as acellular protein-based scaffolds, provide structural support and facilitate growth factor delivery. Although these approaches have demonstrated promising preclinical and clinical outcomes, limitations such as incomplete restoration of skin appendages, insufficient vascularization, high cost, and poor host tissue integration continue to restrict their widespread clinical translation. More recently, 3D bioprinting scaffolds have emerged as an advanced strategy offering precise control over scaffold geometry, porosity, and bioactive distribution. Unlike conventional hydrogels or nanofibers, bioprinting enables the fabrication of patient-specific constructs incorporating cells, growth factors, and antimicrobials in defined architectures, resulting in improved angiogenesis, accelerated wound closure, and reduced inflammation in preclinical studies (Primous et al., 2024).

#### 4.1.1 Nanofiber scaffold

Electrospun nanofibers from natural and synthetic polymers deliver antimicrobials, growth factors, or antioxidants directly to the wound. Examples include chitosan- PVA (polyvinyl alcohol) nanofibers with zinc oxide for antibacterial action, and Poly (lactic-co-glycolic acid) (PLGA) scaffolds with antibiotics or growth factors to stimulate angiogenesis. Thus, this approach combines wound protection with targeted therapy, showing strong potential for diabetic wound management. Various antibacterial nanofibers for wound healing were studied by Merazougui et al. (Merzougui et al., 2022). Shinaoka et al. focus on engineering scaffolds that mimic the ECM to restore both structure and function of damaged skin. It reviews biomimetic strategies such as electrospun nanofibers, porous sponges, and hydrogels that replicate ECM morphology and mechanics. Aliyu et al. proposed nanofibrous scaffolds, evaluated as superior alternatives to conventional dressings due to their high surface area, biomimetic structure, and drug-loading potential (Yusuf Aliyu and Adeleke, 2023).

**FIGURE 7:**
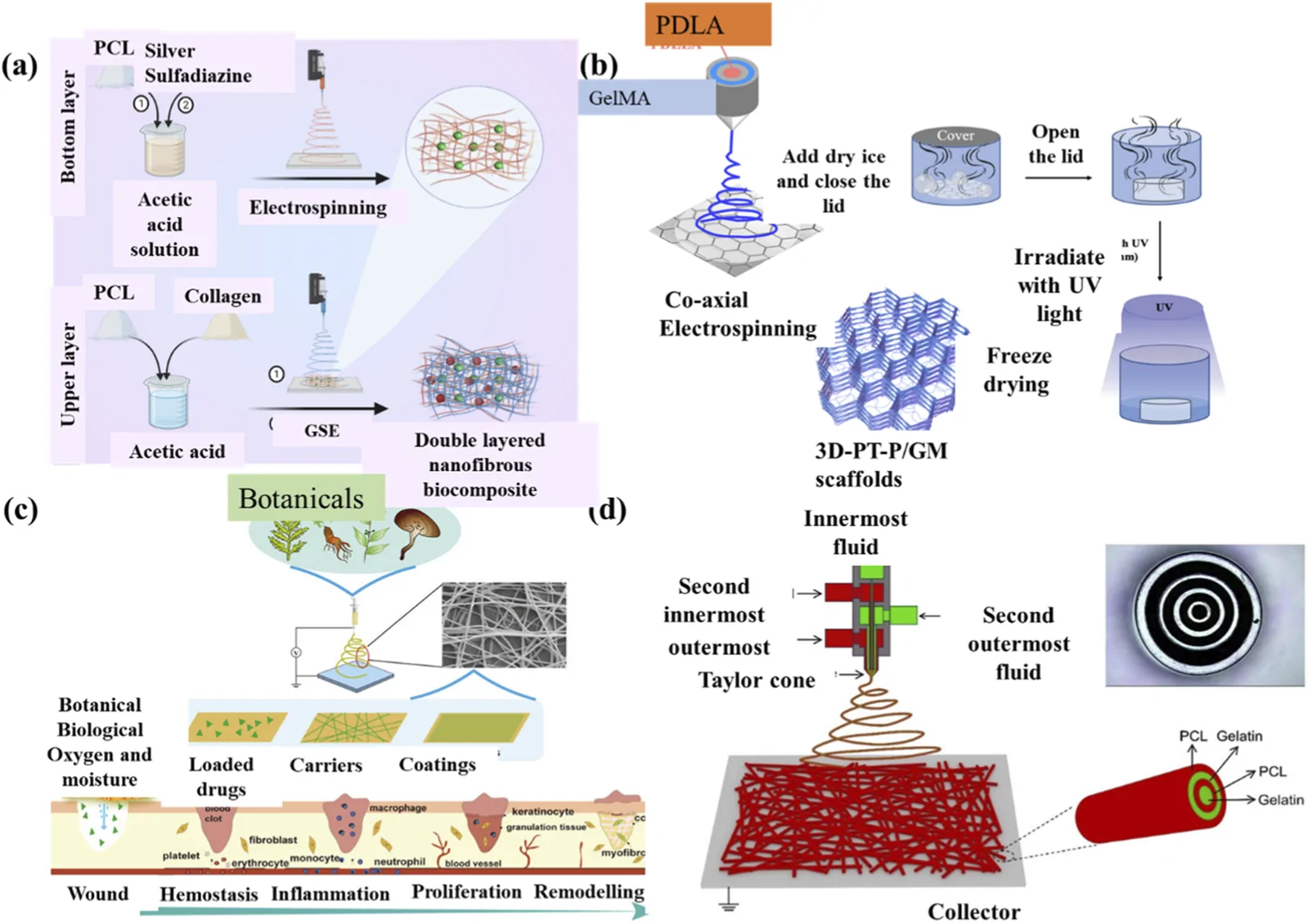
**(a)** Electrospinning representing fabrication of the double-layered nanofibrous scaffold mat with chitosan and polycaprolactone (PCL). Reproduced with permission, copyright 2023, Scientific reports (Nejaddehbashi et al., 2023). **(b)** The preparation process of gelatin methacryloyl (GelMA) scaffold using freeze-drying method. Reproduced with permission, copyright 2022, Springer nature (Li et al., 2022b). **(c)** Electrospinning of botanicals for skin wound healing. Reproduced with permission, copyright 2022, frontiers (Guo et al., 2022). **(d)** Quadriaxial electrospinning, with the inset in the upper right corner displaying a photograph of the concentric spinneret. Reproduced with permission, copyright 2021, Elsevier (Zhang et al., 2021). Abbreviations used in above figure: grape seed extract (GSE).

The roles of scaffold stiffness, surface charge, and pore size in regulating cell adhesion and proliferation are emphasized, along with the incorporation of growth factors and cells to promote tissue regeneration (Hama et al., 2023), Whereas Bagheri et al. focus on chitosan-based nanofibers for the treatment of diabetic ulcers. The unique properties of chitosan such as antibacterial, biocompatibility make it suitable for wound healing (Bagheri et al., 2022). Jiang et al. demonstrated how electrospun nanofibers loaded with bioactive agents (antioxidants, antimicrobials, growth factors) regulate inflammation, promote angiogenesis, and accelerate re-epithelialization, positioning electrospinning as a versatile strategy for diabetic wound healing (Jiang et al., 2024). Figure 7 illustrates representative fabrication strategies employed in the development of advanced wound healing scaffolds and nanofibrous systems. Figure 7a depicts the electrospinning-based fabrication of a double-layered nanofibrous scaffold composed of chitosan and polycaprolactone (PCL), designed to mimic the structural and functional properties of native tissue and support wound regeneration (Nejaddehbashi et al., 2023). Figure 7b presents the preparation of a gelatin methacryloyl (GelMA) scaffold using a freeze-drying method, highlighting the generation of a porous three-dimensional architecture favorable for cell infiltration and tissue integration (Li et al., 2022b). Figure 7c demonstrates the electrospinning of botanical-based materials for skin wound healing applications, emphasizing the incorporation of natural bioactive compounds into nanofibrous dressings to enhance therapeutic efficacy (Guo et al., 2022). Figure 7d shows the quadriaxial electrospinning technique, including the concentric spinneret design, which enables the fabrication of complex multilayered fibers with controlled drug delivery and multifunctional properties for tissue engineering applications (Zhang et al., 2021).

#### 4.1.2 Hydrogel scaffold

**FIGURE 8:**
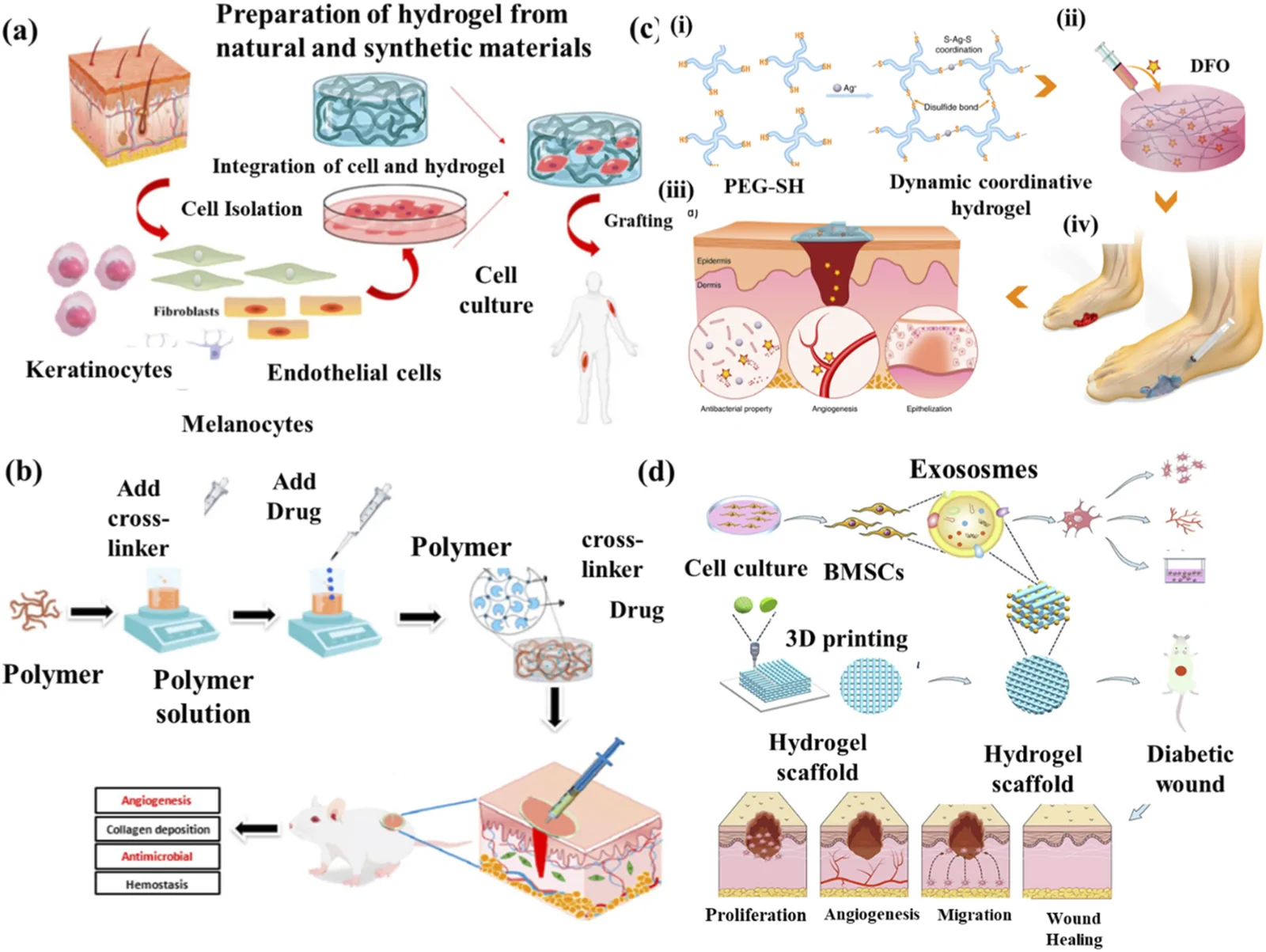
**(a)** Cell isolation for making a bio scaffold in skin tissue engineering. Reproduced with permission, copyright 2024, JSRM (Rezakhani et al., 2024). **(b)** Preparation of hydrogel for wound healing application. Reproduced with permission, copyright 2024, JSRM (Rezakhani et al., 2024). **(c)** (i) Schematic illustration of the self-healing Ag(I)-thiol (Au–S) coordinative hydrogel developed by mixing 4-arm- Polyethylene Glycol (PEG)-SH with AgNO_3_. (ii) *In situ* encapsulation of drug deferoxamine (DFO) to obtain an injectable, self-healing, antibacterial, and angiogenic multifunctional hydrogel for diabetic skin wound repair. (iii) Foot ulcers of type I diabetes (left) and therapeutic effect after hydrogel treatment (right). (iv) Mechanism of the hydrogel in repairing skin defects through injection. Reproduced with permission, copyright 2020, Wiley (Cao et al., 2021). **(d)** Schematic of cryogenic 3D printing hydrogel scaffolds. Reproduced with permission, copyright 2021, Elsevier (Hu et al., 2021).

Hydrogels are highlighted as versatile dressings for diabetic foot ulcers (DFUs), offering moisture retention, oxygen permeability, and bioactive delivery. Güiza-Argüello et al. demonstrate that wounds treated with hybrid hydrogels with nanoparticles, growth factors, or plant-derived agents show accelerated healing in preclinical and some clinical studies (Güiza-Argüello et al., 2022). L. Rezakhani et al. demonstrated natural and synthetic hydrogels for regenerative medicine. In their work, it is proposed that cell-laden hydrogels can be used as grafts for various organs depending on the differentiation of the cells within the hydrogel (Rezakhani et al., 2024). The results emphasize their hydration capacity, biocompatibility, and clinical translation in wound healing, bone, joint, and liver repair, while noting challenges in mechanical strength and long-term integration which has been demonstrated in Figures 8a,b. Figure 8c is an illustration of the self-healing Ag(I)-thiol (Au–S) coordinative hydrogel developed by mixing 4-arm-PEG-SH with AgNO_3_. Figure 8d shows 3D printed hydrogel which can be used as a healing patch on diabetic wounds.

#### 4.1.3 3D bioprinted scaffolds

**FIGURE 9:**
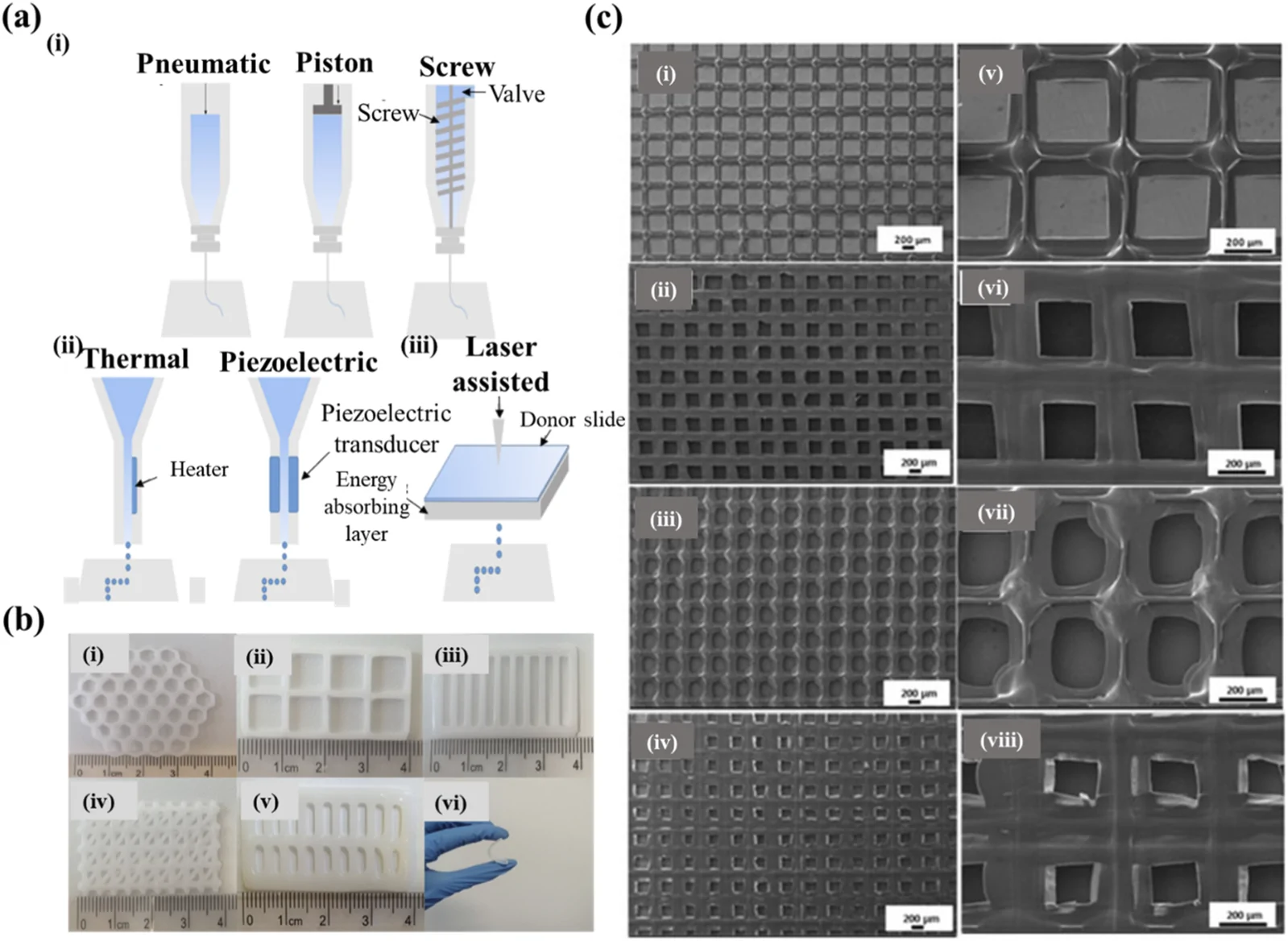
**(a)** Schematics showing (i) 3-D printing techniques an extrusion-based. (ii) inkjet-based, and (iii) laser-assisted bioprinting technologies. Reproduced with permission, copyright 2022, Springer (Glover et al., 2023). **(b)** Digital images showing honeycomb, square, parallel, triangular, double-parallel, and the flexibility of a bioprinted polycaprolactone (PCL) scaffold. Reproduced with permission, copyright 2022, Springer (Glover et al., 2023). **(c)** Scanning electron microscopy (SEM) images of the different 3D printed structures. Reproduced with permission, copyright 2025, MDPI (Harmanci et al., 2022).

Zhong et al. shows a study in which they have developed a 3D bioprinted scaffold using gelatin, decellularized ECM, and chitosan, achieving high antibacterial activity using *S. aureus* and *Escherichia coli* (*E. coli*), good mechanical strength, and proliferation of fibroblast, making it a promising DFU repair scaffold (Zhong et al., 2023). Recent developments include scaffolds infused with growth factors, antimicrobials, or stem cells, which have shown improved angiogenesis, reduce inflammation, and faster wound healing in preclinical studies. Glover et al. demonstrated the benefits of drug-loaded 3D scaffolds for treating DFU. Drug-loaded 3D scaffolds can provide sustained and localized therapeutic delivery, enhance angiogenesis and cell proliferation, reduce infection risk, support extracellular matrix regeneration, and promote faster wound closure in chronic diabetic wounds. The 3D scaffolds with different designs were fabricated for the delivery of the antibiotic, and the characterisation of the scaffolds showed exceptional mechanical properties. This study results are shown have in Figure 9 (Glover et al., 2023).

Figure 9a represents different 3D printing techniques like extrusion based, inkjet based, thermal and laser based which can be used to print scaffolds for wound healing. Figure 9b shows different shapes of scaffolds printed like honeycomb, square, parallel, triangular which are made of polycaprolactone (PCL) polymer. Figure 9c shows the corresponding scanning electron microscopy (SEM) images of the following 3D printed scaffolds.

However, challenges such as achieving vascularized constructs, ensuring mechanical stability, and scaling up production must be overcome before translation. While progress has been made in chronic wound and burn models, complete skin restoration with appendages and full functionality remains a challenge, requiring further integration of mechanobiology and bioactive cues. From the above findings, it can be concluded that, hydrogels provide promising scaffolds for DFU therapy, but clinical translation requires optimization of mechanical strength, infection control, and cost-effectiveness.

### 4.2 Stem cell therapies

Conventional treatments such as debridement, offloading, infection control, and revascularization are often insufficient, with nearly half of patients experiencing ulcer recurrence within less than 2 years (Branski et al., 2009). Considering these constraints, regenerative medicine, especially therapies involving stem cells, has emerged as a promising approach due to its ability to promote tissue healing, blood vessel formation, and immune system regulation (Kosaric et al., 2019). Stem cells are undifferentiated cells that can replicate and differentiate into different types of cells. In wound healing, they act not only by replacing damaged tissue, but also by secreting paracrine factors that regulate inflammation, promote angiogenesis, and stimulate ECM remodelling (Strauer and Kornowski, 2003). Stem cell-based treatment entails extracting stem cells from various sources, including bone marrow, adipose tissue, umbilical cord, or induced pluripotent stem cells (iPSCs), and delivering them to the wound area via local injections, scaffolds, or systemic methods such as intravenous or intra-arterial delivery (Riaz et al., 2025). The therapeutic benefits they provide encompass angiogenesis, neuroprotection, collagen formation, and immunomodulation. Therefore, stem cell therapy extends not only structural repair, but also enhances the microenvironment essential for healing (El Hage et al., 2022).

Various stem cell types have been studied for their possible application in the treatment of DFU. Mesenchymal stem cells (MSCs) obtained from sources such as bone marrow, adipose tissue, umbilical cord, and placenta are the most extensively studied for their ability to differentiate into multiple cell types, their immunomodulatory properties, and the ease with which they can be isolated (Hsieh et al., 2022). Bone marrow-derived mesenchymal stem cells and mononuclear cells (BM-MSCs and BM-MNCs) have been widely used in clinical trials, demonstrating improvements in ulcer healing, enhanced blood flow to limbs, and reduced amputation rates. Kosaric et al. showed BM-MSCs and BM-MNCs derived cells for DFU healing; however, the collection of bone marrow is invasive, and the regenerative capacity of autologous bone marrow cells in diabetic patients is often reduced (Kosaric et al., 2019). Umbilical cord-derived MSCs (hUC-MSCs) have emerged as an attractive allogeneic source, with studies demonstrating their ability to enhance wound healing and limb salvage, particularly when combined with angioplasty to restore circulation. Hu et al. demonstrate the use of iPSCs beyond MSCs is being explored as a theoretically limitless source of patient-specific cells that can differentiate into keratinocytes, fibroblasts, or endothelial cells. While iPSCs hold great promise, safety concerns such as tumorigenic potential remain unresolved. Embryonic stem cells (ESCs) also offer high differentiation capacity, but their application is restricted by ethical controversies and the risk of teratoma formation (Nourian Dehkordi et al., 2019).

**FIGURE 10:**
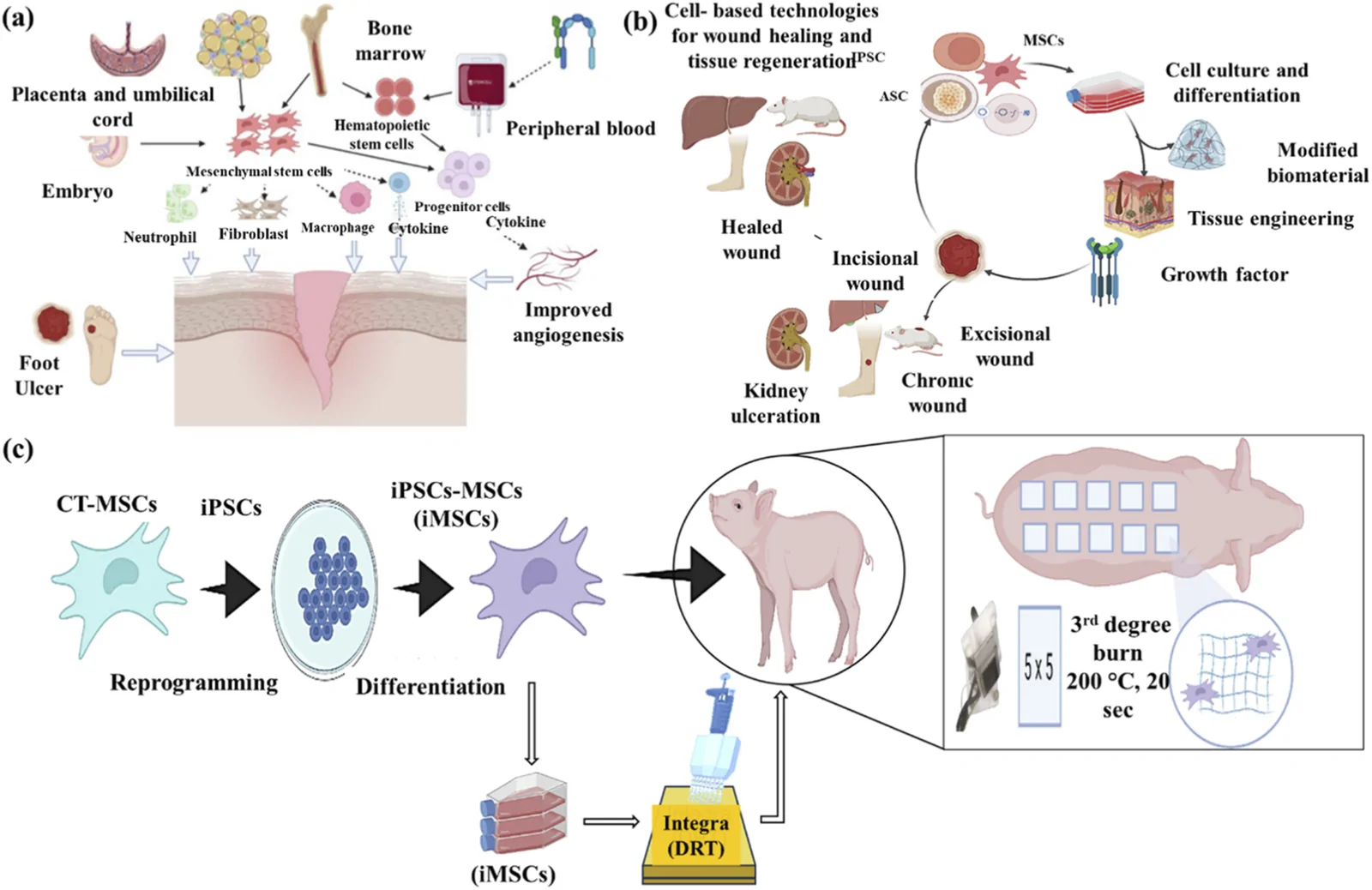
**(a)** Differentiation of stem cells and their therapeutic potential in diabetic foot ulcer (DFU) healing. Reproduced with permission, copyright 2022, MDPI (El Hage et al., 2022). **(b)** Comprehensive workflow of cell-based technologies for wound healing and tissue regeneration. Reproduced with permission, copyright 2025, Nature (Nayak et al., 2025). **(c)** Isolated human connective tissue-derived mesenchymal stem cells (CT-MSCs) were reprogrammed to connective tissue-derived induced pluripotent stem cells (CT-iPSCs) and further differentiated to induced mesenchymal stem cells (iMSCs). These were expanded and seeded onto Integra dermal regeneration template (DRT) for application and testing in our porcine thermal injury model. Reproduced with permission, copyright 2025, Nature (Farahat et al., 2025).

The effectiveness of stem cell therapy for diabetic wounds has been shown through multiple mechanisms. To begin with, stem cells enhance the formation of new blood vessels by releasing VEGF and various other angiogenic factors, which boost the blood supply to ischemic tissues and secondly aid in the delivery of oxygen and nutrients, they modulate inflammation by suppressing pro-inflammatory cytokines such as TNF-α and IL-1β and upregulating anti-inflammatory mediators such as IL-10, thereby restoring immune balance in the wound bed. Third, they enhance collagen deposition and ECM formation, thereby improve the structural integrity and accelerate re-epithelialization. Fourth, they exert neuroprotective effects by releasing neurotrophic factors that ameliorate diabetic neuropathy (Hu et al., 2015). Collectively, these actions not only accelerate wound closure but also improve long-term outcomes by reducing recurrence and lowering amputation rates. Shu et al. proposed a meta-analysis of clinical trials, which confirmed that stem cell therapy achieved significantly higher healing rates compared with conventional therapy (77.4% vs. 31.9%), with additional benefits including reduced pain, improved tissue perfusion, and lower incidence of amputation (Shu et al., 2018). Figure 10a shows the stem cells differentiation from different sources (e.g., embryo, placenta, and umbilical cord, adipose tissue, bone marrow, peripheral blood). The therapeutic mechanism of stem cells in DFU healing includes neutrophil proliferation, fibroblast proliferation, macrophage production, cytokine production, and improved angiogenesis. Figure 10b shows cell-based technologies for tissue regeneration and wound healing. These technologies enhance angiogenesis by increasing levels of VEGF and HGF, and modulating the inflammatory response by reducing levels of IL-1 and TNF-α. Figure 10c shows connective tissue-derived mesenchymal stem cells (CT-MSCs) can be reprogrammed into connective tissue-derived induced pluripotent stem cells (CT-iPSCs), which gets further differentiated into iMSCs and they are used in porcine model having thermal injuries for wound healing studies. Francesco et al. discusses the wide range of clinical applications of adipose-derived exosomes. Adipose-derived stem cells (ASCs) are highly promising due to their abundance, ease of harvest with minimal invasiveness, and strong ability to promote angiogenesis and ECM remodelling (Simonacci et al., 2017).

Even with these promising findings, numerous obstacles hinder the broad implementation of stem cell therapies in clinical settings. A significant issue is the inconsistency in the origin and quality of stem cells. Cells sourced from diabetic patients themselves frequently exhibit reduced functionality, while those from donors may face the risk of immune rejection (Paul et al., 2009). Additionally, ethical and safety considerations limit the use of embryonic stem cells and iPSCs, primarily due to the potential for teratoma formation and ongoing regulatory uncertainties (Li and Li, 2014). Another limitation is the poor survival and retention of transplanted stem cells at the wound site, which reduces their therapeutic efficiency; this has prompted exploration of scaffolds, biomaterials, and gene-modified cells to enhance delivery and engraftment. Furthermore, there is no consensus on the optimal stem cell type, dosage, or administration route, with some studies employing local injection while others use systemic infusion or scaffold-based delivery (Kalou et al., 2021). Cost, scalability, and accessibility represent additional barriers, as stem cell therapy remains expensive and technically demanding. Finally, most clinical studies to date are small and short-term, highlighting the need for larger, multicentre randomized controlled trials to establish standardized protocols and assess long-term safety (Li et al., 2015).

### 4.3 Negative pressure wound therapy

Negative pressure wound therapy (NPWT) delivers controlled sub-atmospheric pressure to a sealed wound bed via foam or gauze, a drape, and a suction pump (Gabriel et al., 2021). The mechanical forces (macro- and micro-deformation), continuous exudate control, oedema reduction, and improved perfusion together stimulate granulation and re-epithelialization, which are the key steps that are often delayed the healing of DFU (Da Silva et al., 2023). Emerging mechanistic data also suggest NPWT can modulate systemic signalling (e.g., circulating microRNAs linked to angiogenesis), and its benefits may extend beyond local biomechanics (Kapusta et al., 2020).

Optimal outcomes are achieved through appropriate patient selection and adherence to standard DFU care principles, including infection control, off-loading, vascular optimization, and treatment compliance, resulting in improved wound-area reduction and decreased amputation or resection risk without increasing overall adverse events (Maranna et al., 2021). Under these conditions NPWT demonstrated superiority over standard moist care for wound closure at 16 weeks in the intention-to-treat analysis. These findings remind clinicians that outcomes depend on appropriate patient selection, adherence, and guideline-concordant application (Wang et al., 2022).

Mechanistic studies have provided further insight into NPWT’s biological effects. Changes in circulating microRNAs linked to angiogenesis have been observed in patients undergoing NPWT, suggesting potential systemic effects beyond local wound healing (Shao et al., 2021). Additionally, preclinical studies comparing NPWT with emerging technologies, such as microplasma therapy in diabetic mice, have shown comparable or even superior re-epithelialization with alternative modalities (Liu et al., 2018).

Overall, current evidence supports NPWT as a valuable adjunct in DFU management, particularly for larger or more complex wounds that require rapid granulation and exudate control [140]. Contemporary wound-care approaches continue to feature NPWT often in combination with biological, dermal substitutes, and digital monitoring reflecting active innovation in protocols and delivery models (Zhang et al., 2020).

### 4.4 Hyperbaric oxygen therapy

In DFUs, inadequate tissue oxygenation is a key factor that hampers healing, as low oxygen levels hinder angiogenesis, granulation tissue formation, and the immune response (Mohsin et al., 2024). Fagalia et al. did a randomized study to show the effectiveness of hyperbaric oxygen therapy (HBOT) as a complementary treatment to standard care, designed to address tissue hypoxia and enhance wound healing (Faglia et al., 1996). The HBOT involves placing the patient in a pressurized chamber where they receive 100% oxygen at 2.0–2.5 atmospheres absolute (ATA) pressure. This procedure leads to dramatically increased concentrations of dissolved oxygen in the plasma, facilitating better diffusion into ischemic tissues (Capó et al., 2023). The therapeutic effects of HBOT in DFU healing are mediated through two biological pathways: reduction of oxidative stress and inflammation. Clinical studies show that HBOT lowers oxidative stress markers such as malondialdehyde, protein carbonyls, myeloperoxidase, and xanthine oxidase, while also decreasing pro-inflammatory cytokines including TNF-α and IL-1βIn addition to growth factors, elevated tissue oxygen tension enhances leukocyte bacterial killing and supports neovascularization. Idris et al. performed local HBOT studies, suggesting that oxygen gradients directly stimulate capillary sprouting and granulation tissue formation in ischemic ulcers. In more advanced cases, HBOT contributes to better skin graft outcomes by increasing oxygen delivery, reducing edema, and upregulating IGF-1, thus improving graft adoption and survival (Idris et al., 2024).

In situations where systemic HBOT is not feasible, local HBOT can be applied directly to the affected limb. Ovchinnikov et al. reported significant reductions in ulcer surface area using local HBOT, suggesting its usefulness as an adjunctive therapy (Pasek et al., 2022). Both systemic and local HBOT are used in combination with standard DFU care, which includes debridement, infection control, offloading, revascularization, and moist dressings when needed. Evidence consistently shows HBOT improves healing and reduces the risk of major amputations when added to standard care (Oley et al., 2024).

## 5 Microfluidics in diabetic wound healing

### 5.1 Microfluidic models for diabetic wound healing

In the last 2 decades, due to the rapid development of micro and nanotechnology, with the integration of chemistry, chemical, mechanical, and biomedical engineering, the new era has emerged to develop lab-on-Chip or micro/nanofluidic devices or micro-total analysis systems (μTAS), which is enabled to perform various biological and biomedical analyses. The microfluidic devices are potentially useful for cellular analysis, drug delivery, drug screening, omics analysis and also for characterization such as optical, mechanical, electrical, and biochemical with the use of minimum sample consumption (Santra and Tseng, 2022).

These devices can potentially manipulate and detect bio samples, reagents, or biomolecules in a microscale environment and precisely perform cellular analysis because of their real-time operations, range of designing, easy fluid control, monitoring, and programmatic switching (Shinde et al., 2018; Kumar et al., 2020).

Microfluidic technology offers an advantage over traditional diabetic analysis by providing real-time monitoring of the wound health to clinicians by using telemedicine systems. This technology helps us to provide rapid detection with small sample volumes, especially with paper and flow-based devices (Pagaduan et al., 2015). For diseases that require quick detection, microfluidic device could provide cheap and disposable diagnostics and would help in early detection. Diabetes alters the concentrations of analytes such as in the insulin, oxygen, and other parameters (Ahmadsaidulu et al., 2024). The evaluation of these criteria can provide an idea of the disease progression. Microfluidic device can be used to measure these analytes and drug testing can be performed on these systems to evaluate if there is any effect on the disease condition. Sokolowska et al. used an islet-on-a-chip 3D model of pancreatic islet cells to study the two classes of lipids which are saponifiable and not saponifiable–palmitic acid hydroxystearic acid (PAHSA). PAHSA has been proposed to show as a potential regulator of glucose-stimulated insulin production and glucagon secretion. These two are the only factors that stimulate the diabetic conditions. This study, however, shows that PASHA has a positive effect on the islet proliferation and enhances glucose stimulated insulin secretion. Thus, qualifying as a potential therapeutic drug (Sokolowska et al., 2022).

**FIGURE 11:**
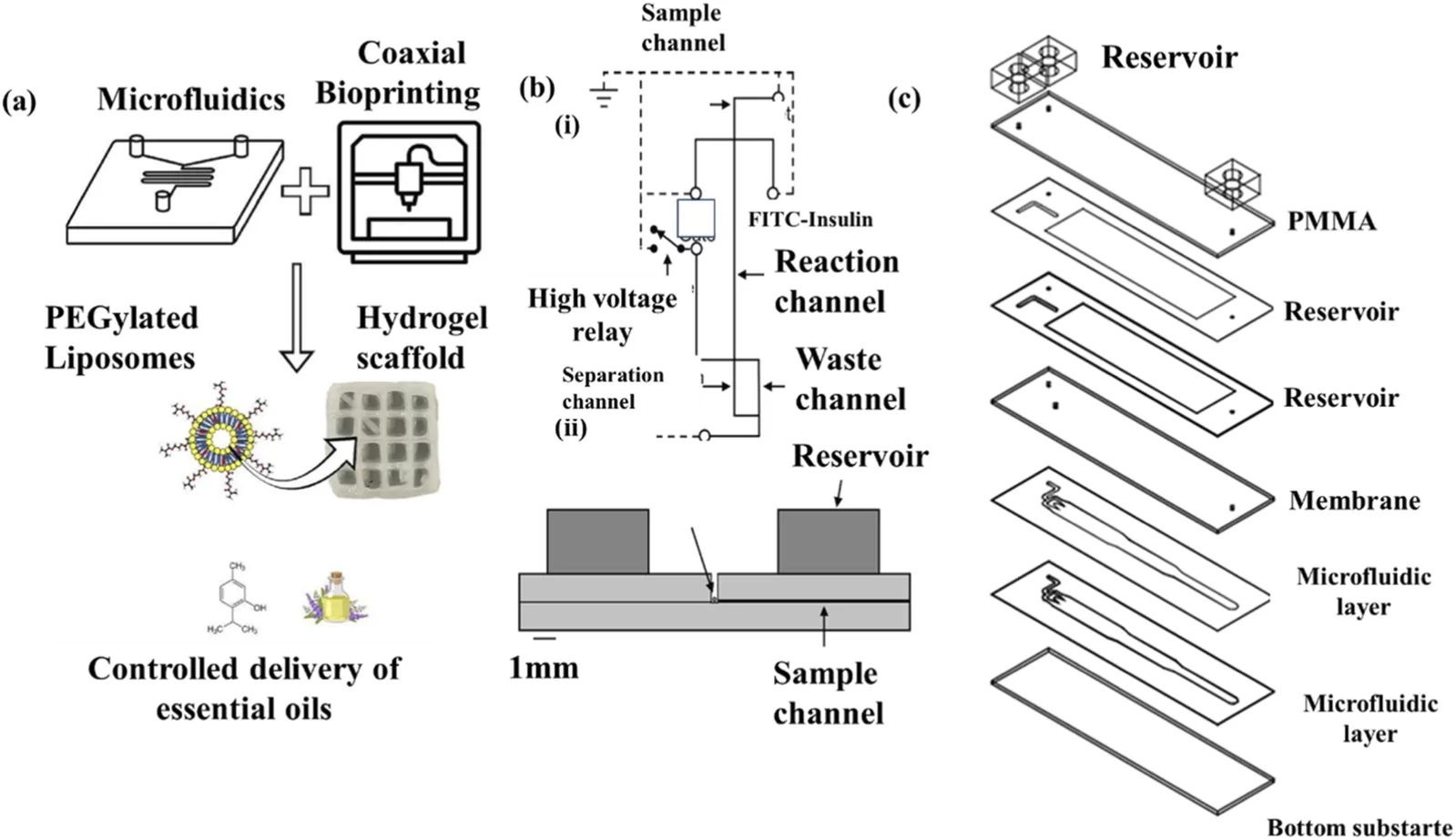
**(a)** Microfluidics and coaxial bioprinting technique used to fabricate a hydrogel scaffold with PEGylated liposomes for controlled delivery. Reproduced with permission, copyright 2003, ACS publications (Fratini et al., 2023). **(b)** Chip layout for a competitive immunoassay and continuous sampling (i) Microfabricated channels (20 μm wide × 3 μm deep) are indicated by solid lines and electrical connections by dashed lines (ii) Side-on, cutaway view of chip at the sampling reservoir. An islet was housed at the bottom of a 300-μm-diameter fluidic access hole. Solution around the islet was sampled by the sample channel. Reproduced with permission, copyright 2023, Elsevier (Roper et al., 2003). **(C)** Layer-by-layer structure of the microfluidic chip with adaptors, 1-mm (poly (methyl methacrylate)) PMMA substrate, 60-μm double-side tape and 260-μm double-side tape. (Reproduced with permission, copyright 2022, Royal Society OF Chemistry (Lin et al., 2019b).

To address hyperglycaemic-related problems in diabetic wounds, Wang et al. proposed a microfluidic device to develop glucose-responsive coacervate protocells. The protocells were formed from diethylaminomethyl-dextran and DNA coacervates, which were coated with phospholipid membranes for stability and biocompatibility. The study confirmed efficient antimicrobial activity, immune modulation, and accelerated wound healing in diabetic mice. This microfluidic chip when integrated with mechanical vibrator are able to generate uniform coacervate micro-droplets via electrostatic interactions (Wang et al., 2024b). Nourmohammadzadeh et al. proposed a three-layer microfluidic array, which uses a hydrodynamic trap to immobilise microencapsulated pancreatic islets for real-time imaging under controlled oxygenation. This device uses a PDMS membrane for dynamic oxygen delivery and simulated hypoxic conditions. Using live-cell imaging, the study shows that hypoxia impairs islet function heterogeneously, altering calcium signalling, mitochondrial energetics, and redox activity. Thus, the device showed significant improvements over conventional hypoxic chambers and offered a high-content, scalable tool for studying cellular physiology in transplantation and disease models (Nourmohammadzadeh et al., 2013). Microspheres are highly biocompatible with tunable mechanics, when combined with a porous scaffold structure that supports fibroblast and endothelial cell adhesion, proliferation, and angiogenesis. Guo et al. developed a microfluidic engineered hydrogel microsphere using Bletilla striata polysaccharide (BSP) integrated with liposome–encapsulated 20(S) - protopanaxadiol (PPD-Lipo@HMS) for diabetic wound healing. In these systems, the microfluidic component typically consists of microscale flow-focusing or droplet-generating channels that produce highly uniform hydrogel droplets under controlled laminar flow conditions. *In vivo* studies on diabetic rat models confirmed accelerated re-epithelialization, collagen deposition, and wound closure, thus confirming the therapeutic potential of herbal nanocomposites fabricated using microfluidics (Guo et al., 2024). Rapid sensing and continuous monitoring of insulin secretion are critical for diabetic wound health insulin secretion from single pancreatic islets using an electrophoresis-based competitive. Hence, Ropar et al. proposed a microfluidic device that continuously monitors immunoassay, which has been demonstrated in Figure 11b (Roper et al., 2003). This chip enabled mixing of insulin, FITC-labelled insulin, and antibodies, followed by rapid secretion and immediate detection. This approach enabled real-time profiling of insulin release with high temporal resolution, capturing both first and second-phase insulin secretion patterns. This can be used in detecting insulin in samples having hyperglycaemic conditions.

Monfared et al. highlight the real-time cell migration analysis using a microfluidic device and integration with molecular stimuli to better mimic *in vivo* healing. These platforms promise improved mechanistic insights into wound closure, angiogenesis, and fibrosis, paving the way for more predictive therapeutic testing (Monfared et al., 2021b). Figure 11a shows integration of microfluidic device and coaxial bioprinting for producing hydrogel scaffold having PEGylated liposomes for controlled delivery of essential oils (Fratini et al., 2023). This showed a proof-of-concept study, which combined the microfluidics and coaxial 3D bioprinting to fabricate multifunctional diabetic wound dressings. Microfluidic device was designed to produce liposomes encapsulating thyme oil, an antimicrobial and anti-inflammatory agent, which were embedded in a hydroxyethyl cellulose hydrogel core, while the outer shell comprised sodium alginate/cellulose enriched with free thyme oil.

A microfluidic-based diabetic wound healing assay was studied using a trypsin flow-focusing to generate controlled wounds of different widths in fibroblast monolayers. In this study, Lin et al. showed that the microfluidic system avoids variability in wound size, such as conventional scratch assays, while enabling simultaneous testing of multiple wounds. The effects of factors, such as shear stress, wound width, and the chemical β-lapachone, on healing were studied. The results showed that wound-healing speed increased with higher shear stress, though linear healing speed remained independent of wound width at low shear stress levels. Figure 11c Shows the stacked microfluidic chip, used in this study. This study showed that β-lapachone does not affect wound-healing rate, thereby highlighting the importance of microenvironment mechanics over chemical simulation (Lin et al., 2019b). Similar studies on trypsin flow and PDMS barriers were simulated in a microfluidic skin-on-chip device, enabling real-time monitoring under physiologically relevant microcapillary shear stress. Gupta et al. experimentally validated that, moderate shear stress enhanced fibroblast migration and wound closure within 24 h, whereas excessive stress disrupted fibroblast adhesion, slowing healing. This helps in the crucial role of microfluidic skin models to replicate *in vivo*-like healing model (Gupta et al., 2022). Microfluidic device is used to synthesis-controlled core-shell alkylated chitosan and calcium alginate microfibers have application in wound dressings, as demonstrated by Xu et al. These fibres support all four stages of wound healing and help rapid blood absorption. It also provides antibacterial and haemostatic activity from alkylated chitosan and supports cell proliferation and tissue remodelling. When loaded with therapeutic agents such as epidermal growth factor, it can accelerate repair. Compared with hydrogel materials, these microfibers offer sequential, stage-specific support for wound healing (Xu et al., 2022). The controlled microarchitecture improved mechanical stability, fluid absorption capacity, and therapeutic loading efficiency, making the fibres suitable for chronic diabetic wound applications (Dey et al., 2025a). Shabestani et al. proposed a rapid prototyped lab-on-chip wound healing assay fabricated by PDMS sheets. This device integrates pneumatic actuators to reproducibly generate wounds in a dermal fibroblast’s monolayer by controlled air pressure (Shabestani Monfared et al., 2020). Wang et al. proposed a microfluidics device that can be used to synthesise porous hydrogel microfibers of prolamins, which are a natural biopolymer for diabetic wound healing (Wang et al., 2024c). Luo et al. tailored the porosity and diameter of the fibres, that can provide flexible drug delivery systems. The encapsulation of therapeutic reagents within these porous fibres resulted in better biocompatibility, sustained release, and enhanced healing efficiency. *In vivo* tests demonstrated superior performance in diabetic wounds testing. Luo et al. proposed a microfluidic device that engineered hydrogel microparticles co-loaded with tea polyphenol-magnesium nanoparticles and melanin nanoparticles for complex wound healing. The spatial separation of the bio-active molecules avoids mutual interference and enhances the stability. These microparticles exhibit enhanced antibacterial properties, and most importantly, effectively scavenging reactive oxygen species, thereby destroying bacterial biofilms and reducing inflammation. Thus, it is very helpful in diabetic wound healing (Luo et al., 2025). Yao et al. show islet cell encapsulation and transplantation cells, that release insulin to restore the insulin levels. This has been demonstrated in studies where microfluidics is used to fabricate thermally sensitive scaffolds made of poly (N-isopropyl acrylamide)/graphene oxide (Yao et al., 2024). Cell-cell interactions, cell migration, and molecular signalling can characterize the usability of the microfluidic systems. Monfared et al. showed that the scratch assay test, in comparison to the microfluidic wound healing assay, has a lack of reproducibility and physiological relevance. Whereas a microfluidic wound assay provides precise spatiotemporal control over environmental factors, such as shear stress, biochemical gradients, and oxygen supply (Monfared et al., 2021a).

### 5.2 Microfluidic systems for drug delivery

To evaluate drug delivery using microfluidics synthesis of nanocarriers, such as metallic, lipids, polymeric, and inorganic nanoparticles, with high precision and high reproducibility is important (Illath et al., 2023a). A microfluidic device allows tight control over particle size, morphology, and drug loading efficiency (Illath et al., 2023b).

**FIGURE 12:**
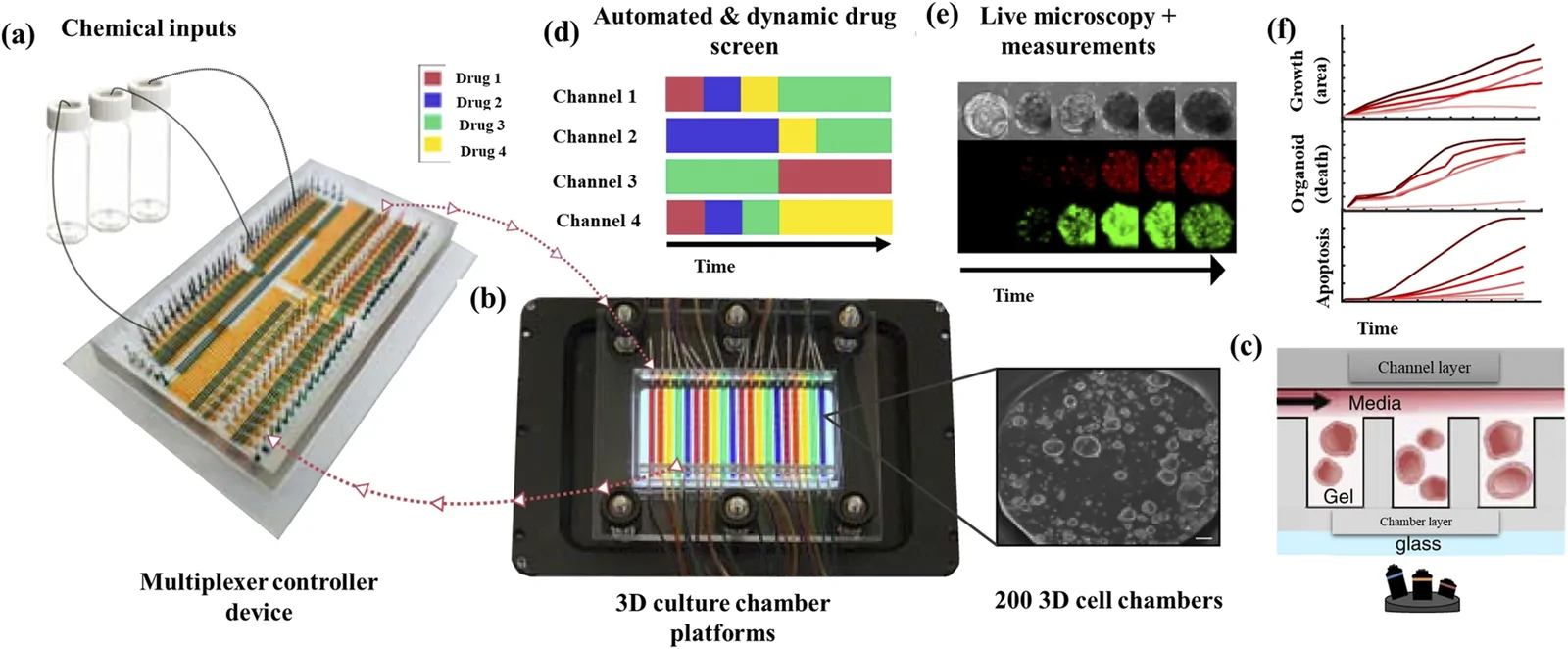
**(a)** A programmable membrane-valve-based microfluidic chip (multiplexer control device) provides automated stimulation profiles to various chambers of a separate 3D culture platform to produce many parallel and dynamical culture experiments. **(b)** The 3D culture chamber platform contains 200 individual chambers that are compatible with temperature-sensitive gels (i.e., Matrigel), and an overlaying channel layer enables 20 independent fluidic conditions (scale bar 100 μm). The channel layer is reversibly clamped on top of the chamber layer to provide media and other chemical stimulation without leakage. **(c)** A cross-section of the two-layer multichambered PDMS-based 3D culture chamber device. **(d)** 30 chemical inputs and 30 outlets of the multiplexer control device **(e,f)** Organoids or 3D cellular structures are continuously observed through time-lapse imaging for quantification; fluidic culture conditions can be changed on demand. The 3D culture chamber device can also be disassembled for cell harvesting and further cellular assays. Reproduced with permission, copyright 2020, nature communications (Schuster et al., 2020).

Prasad et al. proposed a microfluidic device, which can surpass nanoparticle synthesis and enables miniaturised and controlled-size particle synthesis (Prasad et al., 2025). Figure 12a illustrates programmable membrane valve microfluidic chip design for automated fluid handling and control. Figure 12b presents a 3D culture platform containing 200 chambers compatible with temperature sensitive gels along with override channel layer. (Figure 12c) Cross-sectional view of the two-layer PDMS-based 3D culture chamber device. Figure 12d the multiplexer control device includes 30 chemical inputs and 30 outlets. Figures 12e,f shows live imaging and quantification of the spheroids (Schuster et al., 2020). Wang et al. proposed a microfluidic-based polydimethylsiloxane (PDMS) device integrated with microneedle-assisted targeted drug and gene delivery for the treatment of chronic diabetic wounds. The device consisted of a flexible PDMS microfluidic platform containing interconnected microchannels and drug reservoirs capable of transporting therapeutic agents directly to the wound region. The integrated microneedle array enabled minimally invasive penetration into the wound tissue, thereby facilitating localized and controlled release of gene therapeutics and nanocarrier-loaded drugs. The microfluidic architecture allowed precise regulation of therapeutic dosage, flow dynamics, and sustained release kinetics, improving drug retention at the wound site while minimizing systemic side effects (Wang et al., 2025).

Alam et al. proposed a microfluidic device for the synthesis of nanoparticles. The bottom-up approach in microfluidics provides uniformity in nanoparticle synthesis and its bioavailability. Nanogels, or other nanoparticles, enable even greater precision by penetrating deeper into tissues and improving targeting capabilities (Alam, 2023). Delgado-Pujol et al. discussed various administration methods, including topical hydrogels for wound treatment, as well as oral, buccal, injectable, intranasal, and ocular applications. While hydrogels and nanogels have transformative promise, obstacles remain in scaling up manufacturing, assuring biodegradability, and meeting regulatory criteria (Delgado-Pujol et al., 2025). Alaqabani et al. proposed a microfluidic device for hybrid liposomes (HLPs) synthesis. These HLPs were composed of a lipid bilayer and polydopamine (PDA), which conferred pH responsiveness and ensuring that the drug was delivered under acidic conditions, such as those in a diabetic microenvironment, where infection and inflammation are common (Alaqabani et al., 2024). Thus, traditional oral and injectable drug delivery mechanisms are ineffective, and nanoscale delivery systems are more promising, and effective for better drug delivery.

### 5.3 Body fluids for microfluidic glucose detection

Bodily fluids such as blood, interstitial fluid, saliva, sweat, tears and urine can be used to detect glucose using microfluidic device.

#### 5.3.1 Blood

Gold standard method to detect and monitor diabetes is blood glucose test. Finger prick method can be used to take human blood sample and load it in microfluidic devices to measure the glucose concentration in blood (Park et al., 2019). Diabetes can be diagnosed using microfluidic based glucose assay and also based on chemicals such as triglyceride and cholesterol (Li et al., 2022a).

#### 5.3.2 Interstitial fluid

Blood collection can cause scar and loss of finger sensation if continuous blood is being drawn to test the blood glucose level. Researchers have developed minimally invasive microneedle-based systems to draw interstitial fluid and test glucose with that. Takeuchi et al. developed porous microneedle arrays using a salt leaching method that can be used to withdraw interstitial fluid for glucose testing (Takeuchi et al., 2020). Though this method is less invasive but has a lag time of 10 min which attributes to the time taken by the glucose to flow from the bloodstream to the interstitial fluid (Ribet et al., 2018).

#### 5.3.3 Saliva

Saliva collection is neither invasive nor does it have lag time for collection. Various researchers have proposed microfluidic device mostly paper-based device that can readily detect glucose using electrochemical assays (De Castro et al., 2019). Salivary glucose concentrations are relatively high in diabetic patients as compared to a non-diabetic person and thus can be used as an accurate indicator of high glucose concentrations in body.

#### 5.3.4 Sweat, tears and urine

Sweat, urine and saliva are alternate non-invasive body fluids which can be helpful in detecting diabetic conditions. Methods such as colorimetric and electrochemical assays can be used to detect sweat glucose. Allameh et al. and Agustini et al. measured tear glucose using microfluidic paper-based analytical devices (µPADs) (Agustini et al., 2017) with a distance-based colorimetric assay and microfluidic thread-based electroanalytical devices (µTEDs) with an electrochemical assay, respectively (Allameh and Rabbani, 2022).

### 5.4 Organ-on-chip and biosensing

#### 5.4.1 Diabetic wound microenvironments

Biosensors integrated with OOC systems can serve as transformative tools for diagnostics and therapeutics. Compared to the traditional technology in drug testing, OOC integrated biosensor provides sensitive, selective, on-site, and real-time monitoring of the micro-physiological environment. They provide signals for diagnostic and therapeutic applications related to tissue engineering (Adam Kratz et al., 2019).

Dornhof et al. proposed 2-D cell culture models and animal models fail to predict human responses, which leads to high drug attrition rates nearly 90%. The author introduces sensor-integrated OOC platform that enables sensitive, selective, and real-time monitoring of physiological signals, providing dynamic data on how human organs would function in response to a drug (Dornhof et al., 2022). Shinde et al. reviewed different OOC models, such as the heart, liver, kidney, brain, pancreas-on-chip integrated with microfluidics, which can replicate human organ complexity and allow patient-specific drug testing, thereby decreasing reliance on animal models and improving translational accuracy (Shinde et al., 2023).

**FIGURE 13:**
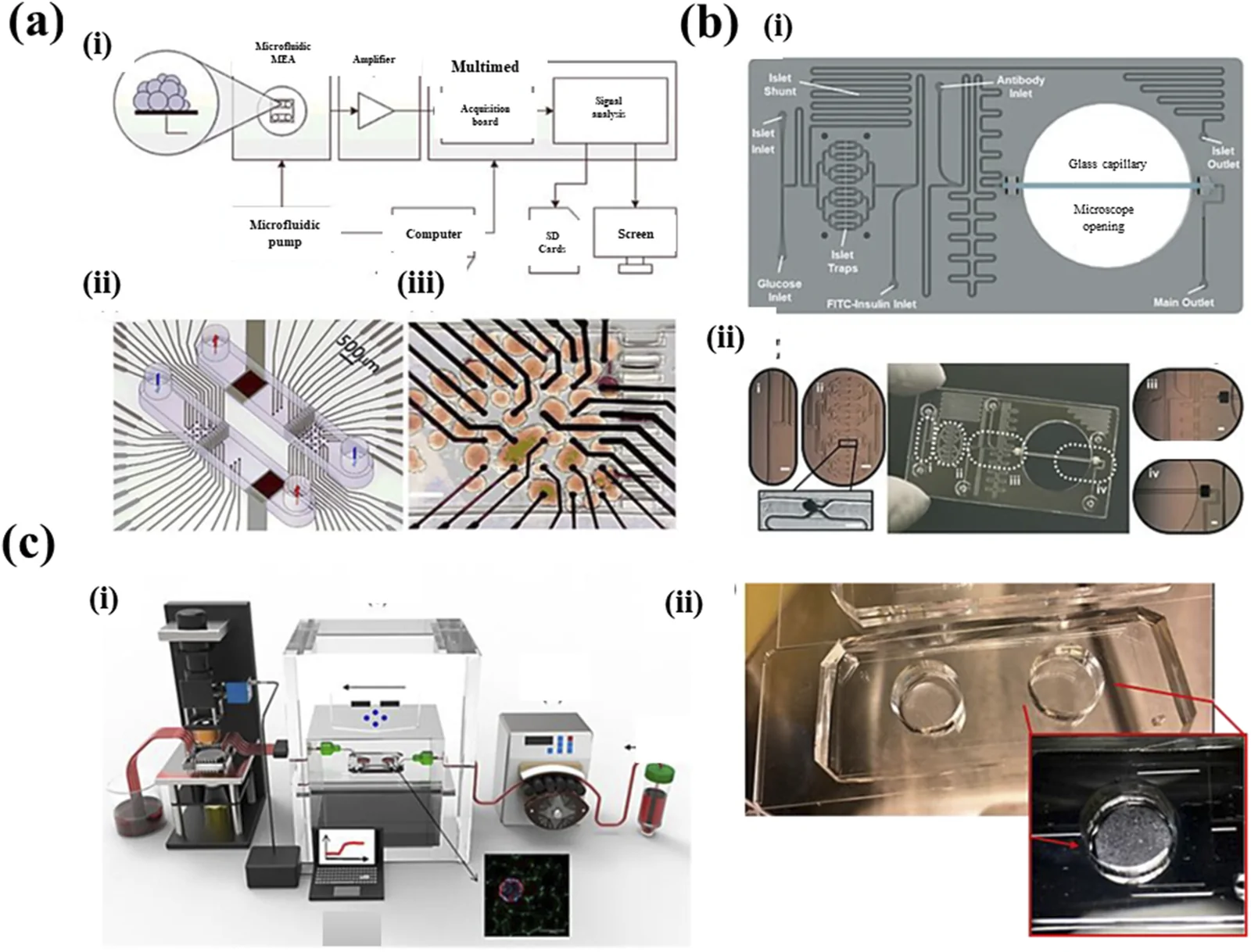
**(a)** Microfluidic-integrated MEA (microelectrode arrays) (i) Layout of the general bench setup for the signal acquisition, analysis, and storage system. (ii) Schematic of the device and the electrode layout. (iii) Microscopic image of islets after seeding on the chip (Scale = 200 μm). (Reproduced with permission, copyright 2023, ACS publication (Shinde et al., 2023). **(b)** Islet-on-a-chip study. (i) Overhead layout of the chip, labelled with channel and trap descriptions. (ii) Image of the fabricated device with optical micrographs of various regions (Scale = 1 mm). (Reproduced with permission, copyright 2023, ACS publication (Shinde et al., 2023). **(c)** LSPR sensing module integrated IOC platform. (i) Schematic layout of the platform and assorted components. (ii) Optical image of the microfluidic device with a zoomed-in view of the islets-embedded carboxymethyl cellulose cryogel structure inside the chamber. (Reproduced with permission, copyright 2023, ACS publication (Shinde et al., 2023).

With the advantages of microfluidic platforms and developments in micro–nano technologies, researchers have been able to conduct numerous comprehensive pancreatic studies. The microelectrode arrays (MEAs) have recently been integrated with microfluidics to study islet activity as explained in Figure 13a. Figure 13b shows islet-on-chip-based studies having glucose inlets, trapping region and mixing area for reagents. Figure 13c shows images of microfluidic device with islets embedded with cellulose cryogel structures. Integration with microfluidics enabled faster online and real-time analysis compared to most earlier MEA systems.

Mittal et al. studied how 3-D organoids, bioprinting, and microfabricated devices, especially PDMS-based OOC systems, can mimic human organs and recreate dynamic tissue environments for biomedical research (Mittal et al., 2019). Clarke et al. proposed a comprehensive review on the advancement of sensor-integrated OOC) devices, focusing on how integrated biosensors improve the functionality and real-time monitoring capabilities of microfluidic systems. The authors highlighted that conventional OOC platforms mainly relied on endpoint analyses, which limited continuous observation of cellular responses and tissue behaviour. To overcome this limitation, the review discussed the incorporation of integrated mechanical, electrical, electrochemical, oxygen, pH, and metabolite sensors into OOC platforms for real-time and non-invasive monitoring of physiological processes. (Clarke et al., 2021). Gao et al. proposed a microfluidics system that integrates cold atmospheric plasma (CAP) treatment with multi-parameter *in situ* sensing to study wound healing in real time. The system enabled simultaneous monitoring of keratinocyte and fibroblast behaviours, including migration, proliferation, and metabolite dynamics under plasma exposure. The platform uses optical, electrochemical, and fluorescence sensors to capture spatiotemporal variations in metabolites, such as nitrite, and in key wound-healing markers, such as keratin KRT14 (Gao et al., 2025). These platforms are extremely useful for drug testing and therapeutic screening. With embedded sensors, we can directly observe how a drug influences glucose metabolism, angiogenesis, or inflammation within a controlled diabetic microenvironment. This improves predictive accuracy compared to 2D cultures and reduces reliance on animal models.

#### 5.4.2 Wearable biosensors integrated organ-on-chip devices for DFU monitoring

**FIGURE 14:**
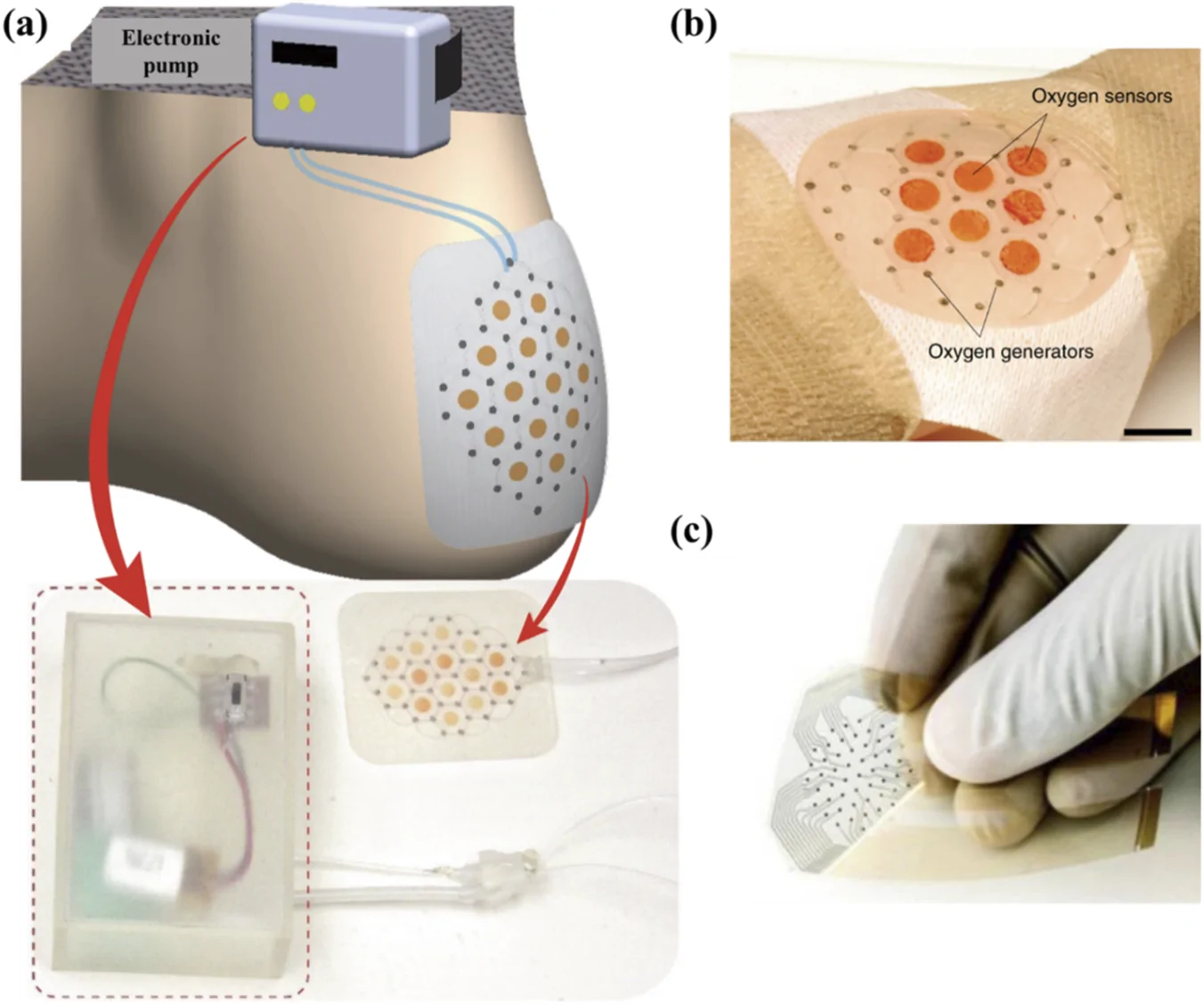
**(a)** Smart wound patch used in DFU application. Reproduced with permission, copyright 2020, nature (Ochoa et al., 2020). **(b)** Pictures showing close-up view of a single patch with oxygen generation and sensing sites. Reproduced with permission, copyright 2020, nature (Ochoa et al., 2020). **(c)** Flexible impedance sensor made via Inkjet Printing. Reproduced with permission, copyright 2018, MDPI (Gianino et al., 2018).

Smart wound patches can provide real-time readings of the wound health parameters. Smart patches can be incorporated with Bluetooth system to transmit data for not only real-time sensing but also real-time data analysis as well. Reza et al. developed a flexible wearable biosensing patch (CW-care patch), which combines an electrochemical sensor array with SU-8-based microfluidic channels to provide real-time wound monitoring. The patch detects metabolites (glucose, lactate, uric acid), ions (Na^+^, K^+^), pH, and temperature. Arrowhead-inspired micropatterns improve fluid-collecting efficiency. Figures 14a,b shows the device integrated with seven sensors for the detection using a gold nanowire-reduced graphene oxide composite device, which enhances sensitivity, while a pH and temperature-correction algorithm maintain accuracy. Wireless data transfer to a mobile application enables ongoing wound monitoring. *In vivo* rat wound model testing confirmed stability and excellent efficiency, indicating that, this system represents a promising advancement in multimodal chronic wound care (Reza et al., 2024). Li et el. Proposed the development of smart wound dressings that go beyond standard hydrogels, foams, and hydrocolloids by incorporating biosensors, drug delivery systems, and adaptive wound responses. Smart dressings refer to advanced wound care systems capable of sensing biochemical and physiological changes in the wound environment, they also respond accordingly through controlled drug release. Smart dressings monitor parameters such as oxygen, pH, temperature, and infection status, enabling real-time data transmission and individualized therapy. The research not only emphasizes clinical potential in minimizing dressing changes, enhancing treatment efficacy, and enabling telemedicine-based wound management, but also discusses constraints such as cost, integration issues, and patient compliance (Li et al., 2024). Specifically, the high costs of fabrication and sensor integration may limit large-scale clinical translation; however, these could be mitigated through low-cost flexible substrates, scalable manufacturing approaches, and printable electronics. Integration challenges in combining biosensors, microfluidics, and wireless components within a compact dressing may affect device stability and signal accuracy, which could be addressed through improved material compatibility, modular architectures, and robust encapsulation strategies. These constraints can be mitigated by using light weight and flexible substrates and designing the device to overcome patient compliance.

Das et al. describes an AI-powered microfluidic devices which are embedded with sensors that can monitor wound variables such as temperature, pH, wetness, and bacterial activity through embedded biosensors. Unlike traditional passive dressings, these microfluidic devices collect the fluid from the wound surrounding and the sensors measure the parameters which are used by AI algorithms to assess data in real time and release medications autonomously through a wireless module for remote monitoring by healthcare personnel. The microfluidic components enable controlled collection, transport, and analysis of wound exudate for real-time monitoring of biomarkers such as pH, glucose, temperature, and infection-related indicators. In addition, microfluidic channels facilitate localized, and stimuli-responsive therapeutic delivery, thereby improving wound monitoring efficiency and enhancing chronic wound management. Preliminary findings indicate improved infection control and faster healing times. The bandage shows promise for clinical use, home care, military medicine, and even space missions, providing a cost-effective, self-regulating, and adaptable wound-management solution that bridges the gap between passive and intelligent wound care (Das et al., 2025). Figure 14c shows the device with microparticle laden and the device integrated with a flexible sensor made with inkjet printing. It underscores that the present wound assessment is subjective, relying heavily on clinical expertise, and that existing dressings (hydrogels, foams, alginates, and silver-based films) lack integrated diagnostic capabilities like real time assessment of the parameters of the wound. Smart dressings with biosensors for real-time monitoring of indicators such as exudate levels, infections, and tissue regeneration may enable tailored treatment regimens. The author demonstrated that combining diagnostic sensors with therapeutic biomaterials is critical for minimizing infection progression, increasing healing results, and lowering healthcare expenditures (Gianino et al., 2018). Mishra et al. proposed the device for the development of wound care. It focuses on theragnostic dressings, which use biosensors and biomarker-responsive drug delivery to monitor wound pH, temperature, glucose, uric acid, and enzymes in real time. Customization and remote care are enhanced by emerging technologies such as electronic skin, 3D printing, and wireless interface integration (Bluetooth, RFID, and NFC) (Mishra et al., 2024). Notwithstanding these developments, challenges still exist with clinical adoption, long-term performance, and cost-effectiveness, highlighting the necessity of translational research to integrate these cutting-edge dressings into standard practice.

Recent advancements in wound care technologies have led to the development of smart microfluidic devices integrated with biomaterials, flexible electronics, biosensors, and stimuli-responsive polymers for adaptive and controlled wound healing. These systems enable continuous monitoring of wound biomarkers such as metabolites, ions, pH, temperature, and infection-associated indicators through integrated sensing platforms and microfluidic channels. Furthermore, AI-assisted microfluidic devices combine real-time biosensing with automated therapeutic delivery and wireless monitoring, thereby supporting personalized, remote, and resource-efficient chronic wound management.

## 6 Limitations and future prospects

Microfluidic devices have emerged as a translational tool for monitoring diabetic wound healing and wound health, offering a highly patient-specific platform to address the limitations of conventional *in-vitro* and *in-vivo* approaches. Traditional models, such as trans-well migration and scratch assays, give fundamental insights but fail to provide the *in-vivo* complexity and variations of biochemical heterogeneity of a diabetic wound (Dewangan et al., 2017). Hence, the drawback of these traditional methods, is that they are insufficient in mimicking *in-vivo* conditions, leading to a lack of reproducibility of fluctuating glucose levels, oxygen stress gradients, and they do not take into consideration the immune-endothelial-fibroblast interactions that are important for monitoring tissue regeneration. These drawbacks can be somewhat overcome in animal models, however pose challenges such as species-specific immune responses and wound-healing mechanisms that differ from human wound healing. As a result, translation from animal models to human clinical trials is significantly less. Microfluidic and organ-on-chip platforms integrated with biosensing provide a specific wound environment, cell-cell interactions, and allow spatiotemporal control of ROS levels, cytokine exposure, and oxygenation (Pagaduan et al., 2015). This approach can provide therapeutic screening, wound management, and a controlled microenvironment along with real-time biochemical analysis. Microfluidic wearable devices offer several advancements for diabetes management by enabling continuous, minimally invasive, and real-time monitoring of biomarkers such as glucose, lactate, pH, electrolytes, temperature, and inflammatory mediators in biofluids including sweat, interstitial fluid, saliva, and wound exudate. These systems improve diabetes control by facilitating early detection of wound infection, monitoring of tissue metabolism, assessment of healing progression, and personalized therapeutic intervention. Integration with wireless electronics and telemedicine platforms further supports remote patient monitoring and timely clinical decision-making (Mishra et al., 2024). Thus, this approach can be a better fit than traditional *in-vitro* and *in-vivo* methods. The level of microenvironment control and reproducibility cannot be achieved with conventional methods, making microfluidic platforms important for therapeutic screening in chronic/diabetic wound healing. Microfluidic based biosensing chip are highly sensitive and will give real time monitoring for relatively low concentrations of the analyte.

However, the fabrication of microfluidic and organ-on-chip devices poses a hindrance to future aspects. The fabrication technique requires multistep lithography, substrate bonding, and surface treatment, which reduces reproducibility and increases the cost and complexity of the fabrication. On the other hand, integrating sensors, valves, pumps, and electronic monitoring systems requires advanced technical skill. Thus, many microfluidic devices remain confined to the engineering stage and struggle to reach clinical applications (Dey et al., 2024). In addition, the long-term stability of the sensors, the reproducibility of the signals, reduced sensitivity, biofouling, and degradation of aptamers/antibodies for electrochemical sensing make the incorporation of the sensors into the device difficult. The clinical implementation of the microfluidic device poses challenges, including reliability, regulatory approval, and robustness, given the complex interactions among hormones, metabolites, ROS, and inflammatory cytokines in diabetic wound.

Future studies may also focus on autonomous closed-loop therapeutic systems capable of both sensing and treatment. These platforms could automatically respond to wound conditions by triggering stimuli-responsive drug release, antimicrobial delivery, oxygen generation, or electrical stimulation based on continuously monitored biomarker levels (Dey et al., 2025b). Standardised fabrication methods, scalable manufacturing technologies, and automated microfluidic assembly systems will improve reproducibility and facilitate large-scale clinical translation.

## 7 Conclusion

Despite challenges in standardization and scaling, microfluidic, organ-on-chip integrated with biosensing platforms are presented as promising tools to bridge the translational gap and support more accurate preclinical screening. These approaches not only reduced experimental variability and labour, but also offer non-invasive, multiparametric insight into organ physiology. The devices have the potential to accelerate drug screening, reduce animal testing, and enhance tissue engineering by dynamically evaluating stress, strain, and biomarker release in living microenvironments. These systems improve the fidelity of *in vitro* and *in vivo* wound models by mimicking the dynamic conditions of native skin repair. The devices are capable of advanced strategies such as stimuli-responsive release, organ-specific targeting, and spatiotemporal control of drug delivery. Microfluidic and organ-on-chip-based sensing devices improve the fidelity of *in vitro* and *in vivo* wound models by recreating key physiological features of the native wound microenvironment, including controlled fluid flow, nutrient and oxygen gradients, cell–cell interactions, and mechanical stimulation. These platforms also enable continuous real-time monitoring of wound biomarkers and therapeutic responses, thereby providing a more accurate representation of wound healing dynamics compared with conventional static culture systems. Microfluidic platforms integrated with advanced biosensors and patches for wound healing, as well as real-time wound monitoring, have potential for clinical trials in the future with regulatory approval. Challenges include calibration in real exudate, durability, data reliability, cost, and regulatory validation, all of which are needed to draw attention in the future. These microfluidic platforms offer particular promise for diabetic foot ulcer management, where non-invasive, real-time monitoring of inflammation, infection, and healing biomarkers could enable earlier intervention and reduce amputation risk. Organ-on-chip models recapitulating the hyperglycaemic, hypoxic microenvironment of diabetic skin may improve preclinical screening of DFU-targeted therapeutics.
