# Supplementary material for: Microfluidics, 3D bioprinted scaffold and organ-on-chip with biosensing activated therapeutic strategies for diabetic wound healing
Source: Front Bioeng Biotechnol. 2026 Aug 7;14:1865992. doi: 10.3389/fbioe.2026.1865992 (PMC13493549; doi:10.3389/fbioe.2026.1865992)
Supplement: Supplementary file 1 [file Supplementaryfile1.docx]

**Microfluidics, 3D Bioprinted Scaffold and Organ-on-Chip with Biosensing Activated Therapeutic Strategies for Diabetic Wound Healing**

S1. .Normal wound healing process.

## 1. **Normal wound healing process**

Traditional wound healing is recognized as a highly intricate and tightly regulated physiological process aimed at restoring both the structural framework and functional integrity of the skin after injury (Kirsner et al., 1993). This repair mechanism is governed by a dynamic interaction of cellular activities, molecular mediators, and biochemical pathways, which are systematically organized into four overlapping yet distinct phases: haemostasis, inflammation, proliferation, and remodelling.

**1.1. Haemostasis phase**

The haemostasis phase begins immediately upon tissue injury to prevent excessive blood loss (Singh et al., 2017). This phase is characterized by vasoconstriction and the activation of platelets, which aggregate at the site of the wound and initiate the coagulation cascade, in which exposure of tissue factor activates the extrinsic pathway, resulting in thrombin generation and the conversion of fibrinogen to fibrin (Green, 2006). The end outcome is a combination of a durable fibrin clot made of cross-linked fibrin, fibronectin, and vitronectin, which not only provides a physical barrier to bleeding, but also serves as a provisional matrix for cell migration (Falanga, 2005). When the platelets are trapped within the clot, they secrete a broad range of growth factors and cytokines that actively promote tissue repair. These include platelet-derived growth factor (PDGF), transforming growth factor-β1 (TGF-β1), epidermal growth factor (EGF), and thrombospondins (Bi et al., 2026). Collectively, these bioactive molecules play a crucial role in conditioning and priming the wound environment, thereby facilitating the progression to the subsequent phases of the healing process (Jeffcoate et al., 2004).

**1.2. Inflammatory phase**

The inflammatory phase begins within hours of injury and is essential for the removal of damaged tissue and protection against infection. Neutrophils are the first responders, recruited via chemotactic signals such as interleukin-1β (IL-1β) and TNF-α (Reza Amini et al., 2020). These immune cells perform antimicrobial activities by engulfing pathogens through phagocytosis, producing ROS, and releasing proteolytic enzymes during degranulation. (Balakrishnan et al., 2025). Neutrophils also create neutrophil extracellular traps (NETs), which are made up of granular proteins, and chromatin, and aid in capturing and neutralizing pathogens.

Macrophages succeed neutrophils at the wound site and are key modulators of the healing response (Velnar et al., 2009).Initially polarized to a pro-inflammatory (M1) phenotype, macrophages secrete growth factors such as VEGF, fibroblast growth factor (FGF), and TGF-β1, and additional cytokines, which further amplify the inflammatory response and recruit other reparative cells (Chen et al., 2023). Once the wound microenvironment reaches a stabilized state, macrophages undergo a shift toward the anti-inflammatory M2 phenotype, which supports tissue repair by stimulating fibroblast recruitment, activating keratinocytes, and facilitating the formation of new blood vessels through angiogenesis (Balakrishnan et al., 2025). The timely resolution of inflammation is crucial, and prolonged inflammation is a hallmark of impaired wound healing (Mahmoud et al., 2024).

**1.3. Proliferative phase**

Fibroplasia, angiogenesis, and re-epithelialization are the processes that restore tissue structure during the proliferative phase (Landén et al., 2016). After being stimulated by PDGF and TGF-β1, fibroblasts migrate into the wound bed and release ECM components, including glycosaminoglycans, fibronectin, and type III collagen. These chemicals interact to form granulation tissue, which promotes the growth of new capillaries and serves as a scaffold for cellular proliferation (Olczyk et al., 2014). The wound bed acquires its distinctive red and granular appearance from endothelial cells that proliferate from pre-existing arteries and form capillary loops. To reestablish epidermal continuity, keratinocytes at the wound borders multiply and move across the granulation tissue at the same time. Angiogenesis, driven by hypoxia-induced VEGF secretion, ensures the delivery of oxygen and nutrients to metabolically active cells in the wound (Ramakrishnan et al., 2014). Endothelial cells sprout from existing vasculature and form capillary loops, giving the granulation tissue its characteristic red, granular appearance. Simultaneously, keratinocytes at the wound margins gradually undergo proliferation and migrate over the granulation tissue, re-establishing the epidermal barrier. This process is mediated by integrin interactions, MMPs, and cytoskeletal remodelling (CLARK, 1993). Integrins such as αvβ5 and α6β4 play key roles in keratinocyte adhesion and motility across the provisional matrix.

**1.4. Remodelling phase**

The last stage of wound healing is characterized by the maturation and structural reorganization of the newly generated tissue. In this phase, fibroblasts differentiate into myofibroblasts under the influence of TGF-β, enabling wound contraction by drawing the ECM together, thereby decreasing the overall wound area (Cialdai et al., 2022). Concurrently, collagen undergoes remodelling through a highly controlled mechanism in which the initially deposited type III collagen is progressively substituted with the mechanically stronger type I collagen, thereby restoring tensile strength (Witte and Barbul, 1997). The turnover of ECM components is finely regulated by the coordinated actions of matrix metalloproteinases (MMPs) and their TIMPs, which maintain equilibrium between degradation and deposition (Łukaszewicz-Zając et al., 2014). As angiogenesis regresses, the capillary density within the tissue decreases, and cellularity in the wound bed declines due to programmed cell death (apoptosis). Although the repaired tissue does not fully replicate the characteristics of intact skin, particularly lacking adnexal structures such as sweat glands and hair follicles, it generally recovers substantial barrier function and mechanical resilience (Baltzis et al., 2014).

In summary, normal wound healing is a tightly regulated process involving a series of cellular and molecular interactions. Each phase is dependent on the successful completion of the previous one, and any disruption in this sequence can result in impaired or chronic wound formation. Understanding these physiological processes provides critical insight into the pathophysiology of chronic wounds and the rationale for advanced therapeutic strategies.
